# Advancing Power Transformer Cooling: The Role of Fluids and Nanofluids—A Comprehensive Review

**DOI:** 10.3390/ma18050923

**Published:** 2025-02-20

**Authors:** Sandra Sorte, Alexandre Salgado, André Ferreira Monteiro, Diogo Ventura, Nelson Martins, Mónica S. A. Oliveira

**Affiliations:** 1 TEMA—Centre for Mechanical Technology and Automation, Department of Mechanical Engineering, University of Aveiro (UA), Campus Universitário de Santiago, 3810-193 Aveiro, Portugal; alexandre.salgado@ua.pt (A.S.); andrefmonteiro@ua.pt (A.F.M.); diogoventura@ua.pt (D.V.); nmartins@ua.pt (N.M.); monica.oliveira@ua.pt (M.S.A.O.); 2 LASI—Intelligent Systems Associate Laboratory, 4800-058 Guimarães, Portugal

**Keywords:** cooling system, insulation oils, thermal performance, dielectric performance, nanoparticles, nanofluids, properties modelling, stability, ageing

## Abstract

The ongoing pursuit of enhanced efficiency and sustainability in power transformer cooling systems has spurred extensive research into the properties and performance of insulating fluids. This review explores the evolution of transformer cooling technologies, focusing on traditional mineral oils and the emerging roles of alternative fluids, such as natural and synthetic esters, and nanofluids. Mineral oils, though widely used, degrade over time, leading to a reduction in breakdown voltage (BDV) from 46 kV to 30 kV, exhibiting low fire resistance. Natural and synthetic esters provide improved biodegradability, fire safety but have higher viscosities—potentially limiting convective cooling. Nanofluids, have demonstrated BDV enhancements of up to 47.8%, reaching 88.7 kV in optimised formulations, alongside increases in partial discharge inception voltage (PDIV) of 20–23%. Additionally, thermal conductivity improvements of 5–20% contribute to enhanced heat dissipation. Moreover, it addresses challenges such as nanoparticle agglomeration, sedimentation, ageing, and compatibility with transformer materials. The analysis provides critical insights into the trade-offs between technical performance and economic feasibility. Concluding with an outlook on future research directions, the review identifies key parameters across various categories, establishing a roadmap for nanofluid integration with existing transformer systems.

## 1. Introduction

Energy underpins modern society, with electricity playing a pivotal role in addressing climate change and environmental challenges [1,2]. Decarbonisation depends on the adoption of sustainable technologies such as solar, wind, hydropower, and biomass, which drive energy electrification [2,3]. However, systemic inefficiencies in energy transformation and distribution networks highlight the need for infrastructure upgrades to optimise integration and ensure efficient energy delivery [4,5].

Rising electricity demands, driven by electric vehicles, smart technologies, and automation, expose significant limitations in existing grids [6]. Modernising energy infrastructure enhances resilience, reliability, and efficiency, supporting the transition to a sustainable and carbon-neutral future [7,8]. A well-designed grid with adaptable and scalable high-voltage transmission systems ensures safe voltage delivery to end-users, minimises energy losses over long distances, and effectively manages increasingly complex electricity flows, which are critical to the stability and efficiency of the power network.

Transformers are critical components of electrical power systems, facilitating electricity generation, transmission, and distribution. They are used at every transition point between voltage levels, making them one of the most strategic assets in power grids. Power systems consist of multiple generation locations, distribution points, and interconnections, and transformers must step up or down the voltage at each point. Transformers are classified based on various criteria, such as power capacity (distribution transformers: 50–2500 kVA; power transformers: above 2500 kVA), insulation type (dry-type or liquid-insulated), and their use in primary or secondary substations [9]. In typical transformer stations, transformers account for nearly 60% of the total investment, underscoring their importance [10].

As electromagnetic devices composed of two or more windings coupled by a mutual magnetic field, transformers are expected to operate reliably and efficiently over many years. One of the main design challenges of transformers is managing the heat generated during operation, as excessive temperatures can damage the insulation and lead to costly failures. The cooling performance of transformers is critical, as it determines how much power can be safely handled without overheating [11]. Given that many transformers worldwide are nearing the end of their designed operational life [12], even as electricity demand continues to rise; proper thermal management has become increasingly important to ensure these essential components’ reliable performance and longevity.

Temperature is a key factor influencing the lifespan of transformers, particularly in oil-immersed models. The heat generated in the windings, primarily due to resistive (copper and iron) losses, can significantly raise internal temperatures [13]. In oil-filled transformers, temperature regulation becomes critical to prevent damage to the insulation system, usually composed of cellulose insulation and liquid dielectric coolant. The hottest point, known as the hotspot in the solid winding insulation, is a crucial factor that determines the operational limits of the transformer [14]. If the temperature exceeds recommended levels, it accelerates the breakdown of the dielectric medium and the ageing of the cellulose insulation, resulting in a shorter lifespan [15]. The deterioration of cellulose insulation is rapid at elevated temperatures.

Mineral oil has traditionally been the preferred insulating liquid in transformers due to its satisfactory dielectric strength, low pour point, and effective heat dissipation [16,17]. However, it presents significant limitations, including low fire resistance, non-biodegradability, and dependence on non-renewable petroleum resources [18]. These drawbacks are increasingly problematic as electricity demand rises and stricter environmental standards are imposed [19].

Research into alternative insulating liquids has been conducted, including silicone oil, natural esters and synthetic esters [20]. These last fluids are biodegradable, fire-resistant, and comply with stricter modern health and safety regulations. Additionally, the incorporation of nanoparticles to form nanofluids is being explored to enhance dielectric, thermal, and cooling properties, further improving transformer performance. For these alternatives to be viable replacements for mineral oil, they must demonstrate robust dielectric strength, thermal stability, low viscosity, chemical resistance, low flammability, and compatibility with existing transformer materials [21,22].

Recent studies have highlighted the potential of nanofluids as advanced insulating materials for transformers, offering promising thermal and dielectric properties. However, existing reviews lack a comprehensive and multidisciplinary approach. Rafiq et al. [23] focused on thermal and electrical properties but overlooked production methods and stability optimisation. Farade et al. [24] analysed the impact of ultrasonication but did not explore broader production challenges or economic considerations. Kalakonda et al. [25] reviewed insulating nanofluids but lacked depth in addressing production scalability, environmental concerns, and practical implementation strategies.

This review adopts a novel multidisciplinary approach to evaluating nanofluids for transformer applications, focusing on the critical thermal, dielectric, and rheological properties required to meet operational and reliability standards. Prioritising these key properties provides a comprehensive perspective on overcoming the challenges of practical nanofluid implementation.

The proposed analysis systematically addresses these challenges: stability and performance, experimental validation, advancing production techniques, and integrating economic and environmental considerations. This unique framework offers a roadmap for discussing the progress of developing optimised nanofluid formulations and enabling their scalable application in transformer systems.

## 2. Methodology

A comprehensive review of the existing literature was conducted using leading international interdisciplinary research platforms, including ScienceDirect, Scopus, and Web of Science. The search employed a strategic combination of the following keywords: power transformer, cooling system, insulation oils, thermal performance, dieletric performance, nanoparticles, nanofluids and properties modelling. The review followed a “semi-systematic” approach, as proposed by [26].

This review provides a comprehensive analysis of the challenges and potential of nanofluids in transformer applications, emphasizing a multidisciplinary approach to addressing existing limitations. It highlights key areas of innovation, including the optimization of stability and performance, advancements in production methodologies, and the integration of economic and environmental considerations.

**Figure 1:**
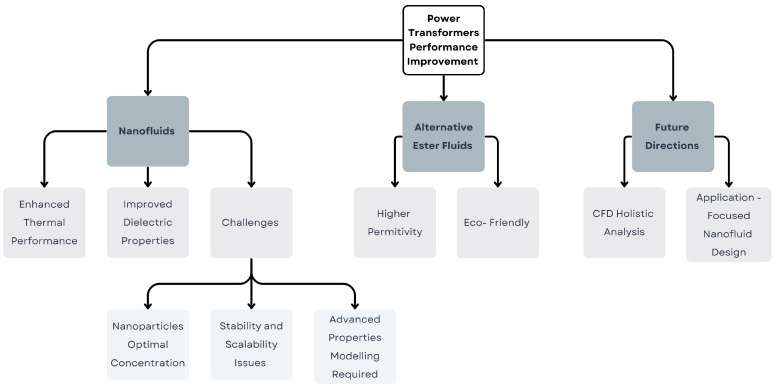
Framework for improving transformer performance via nanofluids, ester fluids, and future-focused designs.

The review includes a comprehensive analysis of literature comprising a total of 100 carefully examined research articles. The article is organized into seven sections: (i) Methodology, outlining the research approach; (ii) Power Transformers Cooling Systems, exploring cooling mechanisms and components; (iii) Fluids for Power Transformer Cooling, discussing types, properties, and modelling; (iv) Nanofluids for Advancing Power Transformer Cooling, analysing the impact of nanoparticles, preparation methodologies, ageing and stability; (v) Challenges and Opportunities for Future Research, identifying unresolved issues and future directions; and (vi) Conclusions, summarizing the study’s key findings and implications. Figure 1 summarizes the findings, presenting a framework for improving transformer performance through three key approaches: nanofluids, ester fluids, and future directions.

## 3. Power Transformers Cooling Systems

Power transformers’ insulation and refrigeration systems are intimately connected to thermal and dielectric performance. Maintaining electrical integrity while preventing breakdowns under high electric fields, a strong function of temperature, is the responsibility of insulation [27]. Meanwhile, the cooling system controls operational temperature to prevent thermal stress from accelerating ageing or affecting insulation dielectric qualities.

The leakage flux around the core of the power transformer (PT) increases when transformer operate beyond rated capacity which in turn increases the temperature of metallic parts and hence changes the thermal dynamics of the system [28]. Elevated temperatures can alter the chemical composition of insulation oil, increasing its gas content and further affecting heat distribution within the transformer.

Additionally, transformer losses, such as ohmic losses in the windings, core losses, and stray losses, contribute to temperature rises. A particularly critical aspect of this thermal behaviour is the hotspot, a localized region within the windings where temperatures peak due to concentrated heat generation and limited dissipation. This non-uniform heat distribution results from current and magnetic flux variations and is influenced by factors such as loading conditions, ambient temperature, and the cooling system’s efficiency. The temperature at the hotspot ultimately dictates the thermal capacity of the transformer, making it a primary consideration in cooling system design [29].

In power transformers, the heat exchange occurs in two main stages. In the first stage, the windings deliver thermal energy to the overlying cooling fluid, usually oil, within the transformer tank. After heating, it is directed to a heat exchanger, which releases energy to the surrounding environment via convection. In this second stage, the heat transfer process can differ for different configurations and the medium used for heat dissipation, where air is the most common fluid [30].

**Figure 2:**
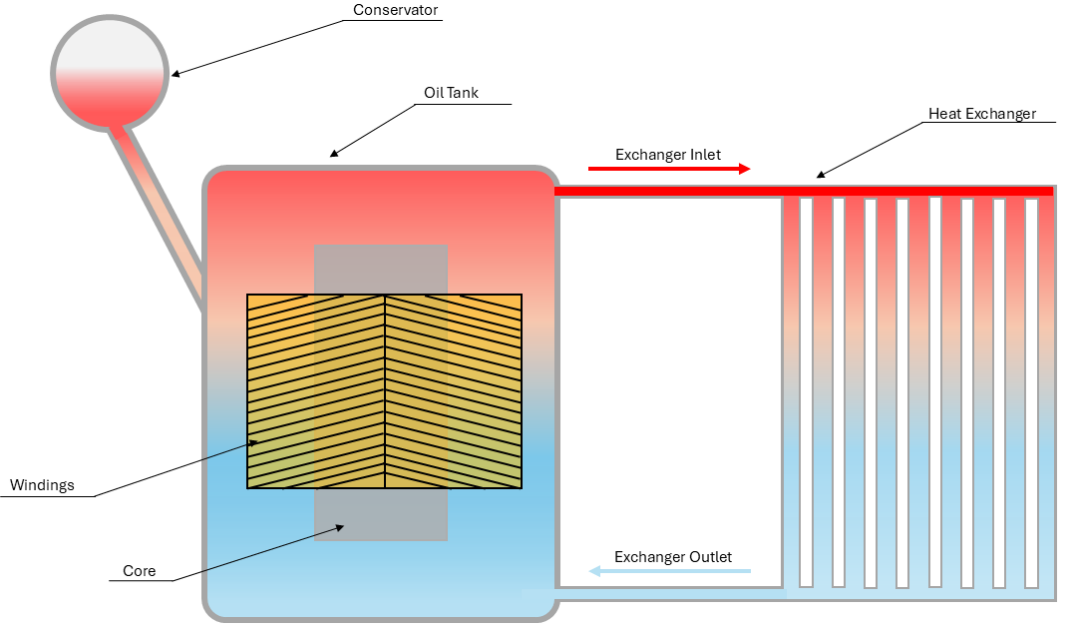
Power transformer refrigeration system—ONAN configuration.

The ONAN (oil natural, fluid natural) configuration is the most straightforward system, as shown in Figure 2, where the cooling fluid and air are conducted through natural convection mechanisms.

As illustrated, the ONAN system works without any pumping assistance and utilizes only density differences created by temperature variation in the oil. The simplicity of this system, along with the removal of many moving components, makes it less prone to failure and requires minimal maintenance [31].

Other categories of cooling systems are ONAN, ONAF, OFAN, OFAF, ODAN and ODAF. The first two letters of these designations indicate the oil (cooling fluid) flow, and the last two the airflow. In both cases, the nomenclature of “N” stands for natural convection and “F” represents forced convection [30]. Considering the scope of this paper, only the variations of oil circulation will be further discussed, namely OF and OD. For representation purposes, they will be presented as OFAN and ODAN.

**Figure 3:**
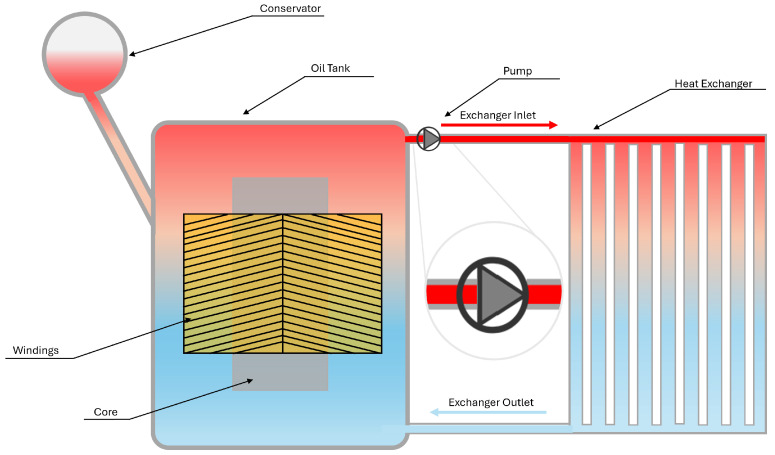
Power transformer refrigeration system—OFAN configuration.

Unlike ON configurations, OF utilizes an oil pump to increase the velocity of oil circulation, as illustrated in Figure 3.

The oil is directed through the transformer following paths of least resistance, therefore, a large amount of oil may bypass the critical active components and flow along the tank walls [29]. This behaviour could limit the oil flow through the windings, even when the pump raises the circulation rates. Furthermore, incorporating a pump introduces additional maintenance demands and raises the risk of system failure.

**Figure 4:**
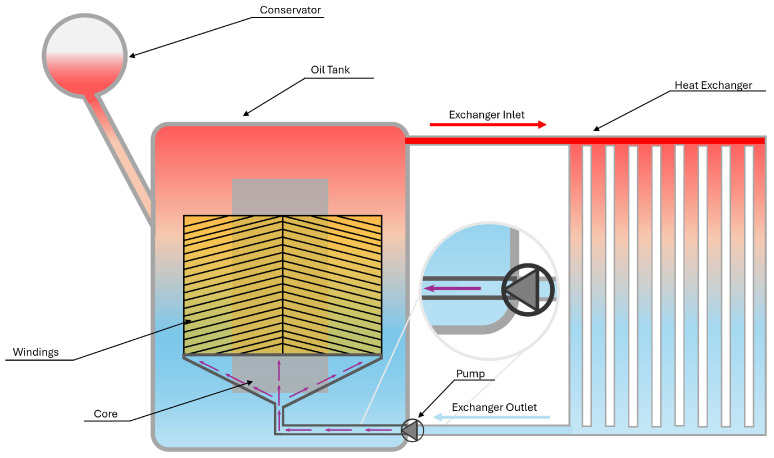
Power transformer refrigeration system—ODAN configuration.

Meanwhile, the OD system, represented in Figure 4, employs internal ducts to channel cooled oil directly into the windings, increasing oil velocity in the active components.

Consequently, oil outside these ducts, such as at the tank’s bottom or lateral sides, exhibits very low velocity, becoming nearly stagnant. While this system shares the pump-related challenges seen in OF systems, it offers the advantage of an extended lifespan due to the improved oil flow explicitly directed toward the windings [32].

Once the dynamics of heat exchange within the external exchanger are understood, examining the internal processes occurring within the transformer tank becomes essential. Transformer oil, a critical component of the insulation system, performs dual roles as an electrical insulator and a cooling agent. The oil fills the transformer tank and permeates the solid insulation, creating a cohesive dielectric barrier across the transformer. It circulates through designated oil channels designed to dissipate heat from active components and ensure temperature stability under varying load conditions [27]. These channels also prevent overheating by allowing continuous heat exchange between the PT and its surroundings.

The solid insulation system complements the liquid component by providing mechanical support and enhanced dielectric strength. It comprises two main elements: thin insulation, primarily Kraft paper wound around conductors, and thick insulation, such as pressboard barriers and spacers. Kraft paper—made predominantly of cellulose (78–80%) with smaller amounts of hemicellulose (10–20%) and lignin (2–6%), is chosen for its mechanical durability and dielectric properties [27]. However, it is thermally sensitive, with a degradation threshold around 105 °C. Advancements such as thermally upgraded Kraft paper (TUK) have emerged, incorporating additives to improve thermal resistance and enlarge the insulation’s service life.

Pressboard barriers divide oil channels into smaller sections, boosting dielectric strength in areas of elevated electrical stress, while spacers support transformer components mechanically and ensure adequate oil circulation. This structural hierarchy reveals the liquid insulation as the weakest element under electric fields. Oils possess a substantially lower dielectric strength than oil-impregnated cellulose materials [33].

The interaction between liquid and solid insulation is crucial for PT longevity but is susceptible to degradation due to chemical reactions such as hydrolysis, pyrolysis, and oxidation [34]. Induced by moisture, hydrolysis processes deteriorate the cellulose in Kraft paper, reducing its mechanical and dielectric properties. Pyrolysis, driven by high temperatures, causes the thermal decomposition of both oil and paper, producing harmful by-products such as gases and acids [35]. Oxidation, the reaction between oil and oxygen, generates sludge and acids, impairing the insulating performance and blocking critical heat dissipation pathways [36].

## 4. Fluids for Power Transformer Cooling

### 4.1. Fluids in Power Transformers

Mineral oil has historically been the standard cooling fluid for PT due to its compatibility with solid insulation and cooling effectiveness. However, its limited oxidation resistance, high toxicity, and poor biodegradability have led to the adoption of alternatives like natural and synthetic esters.

The degree of biodegradability is measured by calculating the lubricant’s conversion rate to $CO_{2}$. A lubricant, hydraulic fluid, or grease is classified as readily biodegradable when 60% or more of the test material carbon is converted to $CO_{2}$ within 28 days, as determined using this test method [18]. According to the study by Arifunnisa et al. the performance of other insulating oils is evident using mineral oil with a biodegradability rate of 30% as a reference [37]. Silicone oil, with only 5% biodegradability, is six times less biodegradable than mineral oil, reflecting its poor environmental profile. In contrast, synthetic esters achieve an 80% biodegradability rate, making them approximately 2.67 times more biodegradable than mineral oil and classifying them as readily biodegradable. With an impressive 99% biodegradability, natural esters are 3.3 times more biodegradable, demonstrating their superior environmental advantages [37].

Natural esters, known for their higher biodegradability than mineral oils, typically exhibit reduced oxidation stability. Studies have assessed their oxidation stability through accelerated ageing tests, measuring key parameters such as acidity and total acid number (TAN), which are crucial for evaluating oxidation resistance [38]. Results consistently rank the of oxidation stability of dielectric fluids in the following order: silicone oil, synthetic ester, mineral oil, natural ester. The increased number of carbon–carbon double bonds in the molecular structure of natural esters contributes to their heightened susceptibility to oxidative degradation.

Esters have proven particularly effective in small and medium-sized transformers, with successful applications in larger units as well [39]. Replacing mineral oil with alternative liquids and completely removing all residual mineral oil is technically impossible during retrofilling. Typically, 4–7% of mineral oil remains in the paper insulation and in other inaccessible areas of the tank. This residual presence results in a mixture of insulating liquids with properties that may evolve during the transformer’s operation.

Nevertheless, substituting mineral oil with ester-based fluids requires careful consideration in transformer design. Esters exhibit over triple the viscosity of mineral oil, slowing coolant flow and heat transfer rates, which may require thermal design adjustments to prevent overheating [40]. Despite this challenge, studies indicate that esters may mitigate cellulose ageing in the insulation system, potentially allowing for higher operating temperatures in ester-filled units [41].

Ester usage also impacts dielectric design. While natural and synthetic esters exhibit dielectric properties comparable to mineral oil under uniform fields, they are more prone to rapid discharges and lower dielectric strength in high-field inhomogeneities or extended gaps [40]. This necessitates modified insulation design to optimize transformer performance when using ester fluids. Esters also have permittivity values approximately 50% higher than mineral oils, promoting a more homogeneous electric field distribution and reducing fluid stress. This is particularly advantageous, as the liquid is often the weakest dielectric component [39].

Both mineral and ester oils degrade over time, reducing breakdown strength, with mineral oil showing more pronounced deterioration due to moisture, acid formation, and fluctuations in power factors. Hao et al. have demonstrated that increased ester content in oil mixtures not only raise breakdown voltage (BDV) but also improves physicochemical properties like dynamic viscosity, acidity, and pour point [42]. The elevated fire and flash points of ester mixtures further enhance their suitability for transformer applications. Synthetic ester mixtures are also known to slow transformer oil ageing, while findings by Dombek and Gielniak suggest that the ester content in oils influences parameters such as relative permittivity, dissipation factor, and net calorific value [43].

Extreme climates, often resembling open-air laboratories, test insulating fluids under challenging conditions. High molecular weight hydrocarbon (HMWH) oils are frequently used in such areas and deliver satisfactory results. Although the pour point of esters is slightly higher than that of HMWH, it was shown by Rapp et al. that freezing natural esters had no impact on their physical and electrical properties, allowing transformers to operate at solid-phase temperatures without adverse effects [44]. The authors have also pointed out that a transformer can be operated at solid-phase temperatures without any detrimental effects.

**Figure 5:**
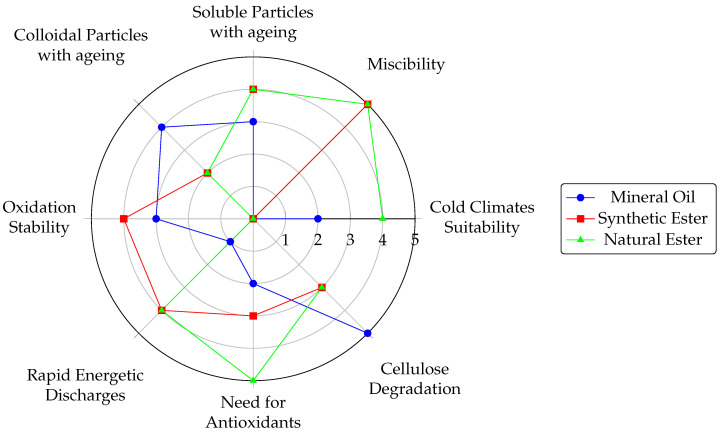
Power transformer refrigeration fluids workability comparison.

Figure 5 summarizes and benchmarks the properties of esters in their natural and synthetic versions against the properties of mineral oil.

To interpret the graph above accurately, consider the following points: The evaluation uses a qualitative, comparative scale from zero to five. Each score reflects the parameter’s significance, whether positive or negative. For instance, a rating of five for absorbance—a less desirable property—indicates high absorbance rather than good performance. Additionally, a score of zero signifies inconclusive research, inconsistent results, or insufficient data.

Antioxidants are essential for preserving the quality and prolonging the service life of transformer fluids by improving their resistance to oxidation. According to the conclusions drawn in the previous paragraph and the studies carried out by Karthik et al, in order for natural esters to have reasonable oxidative stability, it is necessary to incorporate antioxidants [45].

Ageing in oil-paper insulation generates decay products that increase oil viscosity, further hindering heat dissipation. Esters exhibit a greater propensity for viscosity increase due to their acidic content, contributing to sludge formation and affecting insulation quality. Analytical methods like UV spectroscopy provide insights into degradation by detecting sludge and other by-products that reduce dielectric strength. Furthermore, studies on electrostatic charging tendency (ECT) highlights the role of oil composition and ageing effect on dielectric behaviour. Research on palm fatty acid ester (PFAE) mixtures, shows ECT peaks with high PFAE content, underscoring the complexity of oil composition, ageing, and dielectric performance interactions [46].

A finite element analysis of a 280 MVA transformer has revealed significant insights into field distribution and insulation design when using natural esters versus mineral oil. Results suggest that natural ester-filled transformers offer improved field distribution which could also benefit synthetic esters, due to their similar properties. These findings underscore the potential for ester-based mixtures to improve transformer efficiency and longevity through optimized retrofilling strategies [47].

Silicone oils have also emerged as promising alternatives to mineral oils, offering enhanced biodegradability and fire resistance [15]. Studies on their breakdown properties, including radiation exposure from sources like electron beams, X-rays, and photons, highlighting their potential radiation resistance [48]. Furthermore, the electrical resistance of mixtures of silicone liquid with petroleum oils has been investigated, with findings suggesting that they can exhibit significant fluctuations in electrical properties due to the formations of colloids.

Table 1 compares the physical, chemical, and electrical properties of insulating fluids used in power transformers, including mineral oil, natural esters, synthetic esters, and silicone oil [49,50,51].

This compilation provides a structured comparison of essential characteristics, facilitating a clearer understanding of each fluid’s role and significance within the scope of this study. Mineral oil has low density and viscosity, enabling efficient circulation but providing limited thermal and dielectric resistance. Its low flash and fire points indicate higher flammability, while its low acidity and moisture content reduce the risk of corrosion and degradation. However, poor biodegradability makes it less environmentally sustainable. Natural esters have higher density and viscosity than mineral oil, potentially hindering circulation and requiring thermal design modifications. Their high flash and fire points enhance fire safety, and their biodegradability (85–99%) makes them a sustainable alternative. However, higher moisture content and acidity may accelerate degradation and increase maintenance needs. Synthetic esters combine good thermal and dielectric stability with lower viscosity, facilitating a more straightforward application. They offer high breakdown voltage (>75 kV), making them ideal for high-voltage systems, and their biodegradability (89%) adds an environmental advantage. Silicone oil, with the highest density and viscosity among the fluids, provides excellent safety due to high flash and fire points (∼300 °C) and stability under extreme temperatures. However, its lower biodegradability, higher costs and tendency to quick discharges limit its widespread use.

### 4.2. Fluids Properties and Modelling

A comprehensive analysis of the key properties of PT oils that their performance is provided. This discussion builds upon the findings of the previous section and lays the groundwork for subsequent topics. Additionally, the modelling of temperature-dependent properties was explored to offer deeper insights into their behaviour.

The efficiency of insulating oil is primarily characterized by its breakdown voltage, a critical parameter for assessing its performance under electrical stress [52]. Heat transfer within insulating oils is governed by thermal conductivity and convection mechanisms. Convection arises from properties such as viscosity, specific heat, and the thermal expansion coefficient, which drive heat transfer through fluid displacement. At the same time, conduction occurs within the oil’s molecular structure. Additionally, the stability of insulating oil is significantly affected by oxygen presence, with elevated temperatures serving as a catalytic agent for degradation processes [52].

The BDV of an insulating system is influenced by the physicochemical properties of the insulating oil, the presence of impurities, and the configuration of the electrodes. Due to the stochastic nature of electrical breakdown, assessing the reproducibility of experimental measurements requires conducting a sufficient number of tests. Statistical methods derive, representative BDV values, such as the mean or minimum, from the data distribution [53,54,55]. Studies have demonstrated that the BDV of natural and synthetic esters is comparable to that of conventional mineral oil, highlighting their potential as alternative insulating fluids.

The exposure of transformer oil to the environment, such as during leaks or through pipeline systems initiates oxidation processes. This reaction is further accelerated by heat and the presence of dissolved metallic compounds, including iron and copper, which act as catalysts [56]. Additionally, interactions between these oxidation by-products and water can result in internal corrosion within the transformer. The acidity of transformer oil, a critical parameter measured using standards such as ASTM D 974 [57], reflects the extent of degradation.

Acidity, or the acid neutralizing capacity of insulating liquids, arises primarily from oxidation and secondary chemical reactions. Elevated acidity negatively impacts key properties, including the dielectric performance, and contributes to the erosion of cellulose and metallic components, leading to the dissolution of contaminants in the oil. The increase in the acidity rate is a reliable indicator of oil degradation.

The flash point is defined as the minimum temperature at which a substance releases sufficient vapor to form an ignitable mixture in air [28]. Conversely, the fire point represents the lowest temperature at which these vapours sustain combustion for at least five seconds upon exposure to a flame [56]. High flash and fire points are critical for ensuring the safety of insulating oils, with minimum thresholds of 150 °C and 160 °C, respectively, for use in power transformers.

Moisture in liquid insulation can exist as dissolved water or condense into droplets, influenced by equipment temperature and oil condition. Elevated moisture content negatively impacts critical properties, such as reducing BDV, mechanical strength, and dielectric performance. It also accelerates the chemical degradation of the oil and associated paper insulation [58]. The dielectric properties of transformer oils are particularly vulnerable to moisture, compromising the integrity of paper insulation in the transformer core and windings.

Mitigation of moisture-related degradation has been demonstrated with the use of hydrophilic nanoparticles (NPs). Primo et al. showed that incorporating hydrophilic NPs into insulating fluids traps water molecules, preventing the formation of conductive paths that could lead to electrical breakdown in fluids lacking NPs [59].

The solubility of water in insulating fluids increases with temperature, with polar oils absorbing significantly more water across the temperature range [52]. Mineral oil consistently exhibits the lowest moisture content at saturation across all temperature ranges, highlighting its limited capacity to absorb water. In contrast, natural and synthetic esters display considerably higher moisture content at saturation levels. This trend demonstrates that esters are more sensitive to humidity-related issues. While these fluids offer advantages in terms of biodegradability and environmental performance [18], their increased water absorption capacity emphasizes moisture management’s importance in these insulating fluids.

The performance and reliability of insulating fluids are assessed through a range of standardized tests that measure their critical properties. Table 2 summarizes the standard testing methods commonly used to evaluate these properties.

The thermo-physical properties of insulating oil, including density (ρ), thermal conductivity (k), specific heat capacity (cp), and dynamic viscosity (μ), are critical in determining its flow behaviour and temperature distribution within transformer systems. These properties are inherently temperature dependent and nonlinear, which must be accounted for to achieve accurate thermal modelling and analysis. Neglecting this dependency, as it is often done in existing studies, can lead to significant errors in evaluating the oil’s heat transfer performance. Table 3 presents the temperature-dependent expressions of 328 these properties, which are essential for calculating thresholds that integrate these factors 329 to optimize heat transfer and ensure the system reliability.

The outlined relationships provide a comprehensive framework for understanding how temperature variations influence the thermal behaviour of insulating oil.

The miscibility of alternative insulating fluids with mineral oil also plays a is significant role in enhancing the oil’s properties through blending. Natural and synthetic esters are fully miscible with mineral oil in all proportions, making them suitable for mixture formulations. However, when mixed, silicone and mineral oils tend to separate into two distinct layers rather than forming a uniform solution. This is why they are rarely blended directly unless an emulsifier or compatibilizer is used.

Studies by Gockenbach et al. on mixtures of synthetic ester (Midel 7131) and mineral oil (Shell Diala D) revealed that blends with less than 20% ester content maintained electrical and physical properties comparable to pure mineral oil [23,68]. However, at 50% ester content, the density and kinematic viscosity exceeded standard limits. Although density is generally less critical in determining oil quality, it can affect performance in cold climates. Adding ester fluids to mineral oil also reduces the gassing tendency under localized thermal stress.

Miscibility is particularly critical in retrofilling processes. When the old oil (typically mineral) and the new fluid (silicone, synthetic ester, or natural ester) are miscible, retrofilling becomes more straightforward. For immiscible fluids, retrofilling remains feasible but demands greater precision and care to ensure compatibility and performance.

## 5. Nanofluids for Advancing Power Transformer Cooling

Recent advancements in transformer insulation have introduced innovative approaches to enhance the dielectric properties of transformer oils. A notable development is the formulation of dielectric nanofluids (NDFs), achieved by dispersing nanoparticles into transformer oils. Studies in this area focus on improving thermal conductivity and insulating performance [69]. The thermal performance is primarily determined by the thermal conductivity of the nanoparticles within the base liquid, often surpassing that of traditional insulating fluids [69]. Currently, the production cost of nanofluids is significantly higher than that of conventional mineral oils. However, this economic disadvantage could be mitigated if their application substantially improves heat transfer efficiency, enhances durability of the insulation system and extended the service life of the cooling fluid [70].

### 5.1. Nanoparticles and Effects on Fluid Properties

Metal oxide nanoparticles are widely favoured for the formulation of nanofluids due to their affordability, high stability, and ease of production. Examples of common used materials include iron oxides ($Fe_{3}O_{4}$, $Fe_{2}O_{3}$), zinc oxide (ZnO), titanium dioxide $TiO_{2}$, copper oxides ($CuO,Cu_{2}O$), aluminium oxide ($Al_{2}O_{3}$), silicon dioxide ($SiO_{2}$), and boron nitride (BN). These nanoparticles exhibit varying permittivity and conductivity properties: $Fe_{3}O_{4}$, $Fe_{2}O_{3}$, and ZnO are classified as conductive, $TiO_{2}$, CuO, and $Cu_{2}O$ are semiconductive; and $Al_{2}O_{3}$, $SiO_{2}$ and BN are categorized as dielectric materials. Table 4 provides an overview of the most utilized nanoparticles along with their characteristics [23,37,71].

#### 5.1.1. Breakdown Voltage

The relationship between breakdown voltage (BDV), streamer propagation, breakdown time and partial discharge inception voltage (PDIV), in a dielectric fluid (such as transformer oil) is governed by the complex dynamics of electrical discharges. BDV, PDIV, streamer propagation speed, and breakdown time are, therefore, interdependent—controlling streamer formation and propagation can enhance dielectric strength and reliability in high-voltage applications.

Complex charge transport and ionisation dynamics drive the interplay between BDV, PDIV, streamer propagation, and breakdown time in dielectric fluids. BDV and PDIV dictate streamer initiation, while propagation velocity and morphology influence dielectric failure. Nanoparticle dispersion in insulating fluids modifies charge transport, electron trapping, and space charge effects, impacting streamer behaviour and breakdown kinetics. Properties such as permittivity, surface functionalization, and agglomeration dictate these effects, enabling enhanced dielectric strength and reliability in high-voltage systems. These characteristics depend on the nanoparticles’ size, shape, and composition [72].

Numerous studies, particularly those involving mineral oils, have reported that nanoparticle-doped oils exhibit higher BDV levels than pure oils [73]. It is well-established that an increased concentration of water dissolved in insulation fluids significantly reduces dielectric strength. However, the findings indicate that the reduction in BDV caused by dissolved water is less pronounced in nanofluids than in untreated oils [74]. This suggests nanoparticles enhance the dielectric performance of aged insulating oils, which typically exhibit lower BDV than new oils.

The effects of temperature on nanofluids have also been extensively studied. Like pure oils, higher temperatures generally enhance the BDV of nanofluids. Samy et al. revealed that the maximum BDV value depends on nanoparticle size and concentration [75]. Smaller nanoparticles at higher concentrations improved dielectric strength than larger particles, as their larger surface area at the same volume fraction can trap more free electrons [76]. Nonetheless, an excess concentration of nanoparticles can lead to agglomeration and shorter discharge paths, which diminish BDV [77]. This was illustrated in experiments with ${\mathrm{Al}}_{2}O_{3}$ nanoparticles, where agglomeration increased at higher mass fractions, but the use of surfactants mitigated this effect and improved BDV [78].

Discussing particle type, concentration, and size extends beyond the effective enhancement of properties such as BDV. The toxicity of nanomaterials is a prominent area of research, with studies emphasizing the influence of factors such as particle size, surface area, charge, solubility, shape, and crystallinity on biological effects. While dose is traditionally key in toxicity, nanoparticle-specific parameters such as—mass, size, number, and surface area often hold greater significance. Hou et al. identified three primary mechanisms of ${\mathrm{TiO}}_{2}$ nanoparticle toxicity: reactive oxygen species (ROS) generation, cell wall damage and lipid peroxidation caused by nanoparticle attachment, and interactions with biological macromolecules and organelles [79].

Some studies have explored using hybrid particles to leverage the properties of highly toxic particles like ${\mathrm{TiO}}_{2}$ [80]. Combining diverse particles enable the integration of toxic particles with advantageous dielectric properties and non-toxic particles of lesser performance, resulting in fluid with acceptable toxicity levels. Additionally, hybridisation ensures that particles with conflicting thermal and dielectric properties are reconciled, producing a balanced fluid with optimised performance in both domains. Mansour et al. demonstrated that incorporating 0.005 g/L Barium Titanate (BT) nanoparticles with 0.01 g/L ${\mathrm{TiO}}_{2}$ nanoparticles resulted in a 33% enhancement in heat transfer efficiency and a 43% improvement in BDV [81]. Furthermore, the degradation of the dissipation factor was substantially mitigated.

Dielectric strength enhancements in ester-based nanofluids, both natural and synthetic, have also been investigated. Results suggest that at equivalent concentrations, mineral oil-based NDFs outperform their ester-based counterparts in terms of BDV improvement [82,83]. For natural esters, nanoparticle effects on dielectric properties are limited and, in some cases, adverse. Additionally, conductive nanoparticles such as ${\mathrm{Fe}}_{3}O_{4}$, exhibited superior results than insulating nanoparticles like ${\mathrm{SiO}}_{2}$ and ${\mathrm{Al}}_{2}O_{3}$ in specific cases [83]. Table 5 summarizes BDV data for various nanoparticle types, sizes, and base fluids.

The analysis of Table 5 leads to several conclusions. Firstly, it can generally be seen that the presence of nanoparticles contributes significantly to the increase in BDV, with recorded improvements ranging from 1% to 202%. However, the varying experimental conditions—across studies suggest that extreme results should be interpreted cautiously. This reasoning is valid in both directions; even if improvements greater than 100% and those less than zero are considered outliers, the average pattern is still encouraging.

Khaled et al. investigated the impact of ${\mathrm{Al}}_{2}O_{3}$ particles of different sizes dispersed at equal concentration in synthetic ester. Their findings indicate that smaller particle sizes generally results in more significant increases in BDV [91]. Other studies, have confirmed this trend even with different particles and base oils [77,100,105]. However, is not universal that small particles show superior performance than larger particles [92,93]. This suggests that each particle type may have an optimal size for maximum performance, depending on its unique properties.

Regarding concentrations, direct comparisons between low and high concentrations are not straightforward, as average values differ across studies. However, it could be said that it lies within the range of 10–50 nm.

Analysis of the table highlights a close relationship between the binomial particle size and concentration and their combined influence on BDV variation. Specifically, for particles smaller than 50 nm, the most significant BDV increases were observed at concentrations below 0.3 g/L.

Finally, in a broader, qualitative analysis, mineral oil is the fluid with the most significant performance improvements. It exhibits the highest recorded BDV increases and outperforms esters in comparative studies [77]. As far as the particles are concerned, as expected, the semiconductor and conductor particles generally performed better. In the compilation of results conducted, ${\mathrm{TiO}}_{2}$ and ZnO stood out positively. However, since no study has compared all particle types under identical conditions of size concentrations and base fluid, it is not possible to definitively conclude that these particles offer the best performance in terms of BDV enhancement, however it can be said that spherical particles stand out as the most promising ones.

#### 5.1.2. Partial Discharge Inception Voltage (PDIV)

PDIV of nanofluid-based transformer insulation is a critical parameter for determining the reliability and efficiency of electrical systems. By incorporating NPs into transformer oils, nanofluids improve dielectric strength and suppress partial discharges (PD). The effectiveness of this enhancement depends on several key factors, including the type of nanoparticles, their concentration, size, and dispersion stability. Type of NPs are detrimental in what concerns PDIV in nanofluids. Carbon quantum dots (CQD)-coated silica nanofillers, for instance, have been shown to increase PDIV by up to 23% compared to base transformer oil due to their superior electron-trapping capabilities, which suppress PD activity [108]. $TiO_{2}$ and $Al_{2}O_{3}$ nanoparticles also significantly enhance PDIV by forming an electrical double layer (EDL) around the particles, trapping free charges and reducing electron mobility. $Al_{2}O_{3}$ nanofluids impregnated with pressboards, as reported by Atiya et al. [109], exhibit the highest PDIV due to their superior charge trapping. Furthermore, the EDL thickness around nanoparticles plays a decisive role: Atiya et al. [110] demonstrated that a smaller EDL reduces PD activity, as thinner EDLs allow for more efficient suppression of free charge movement.

Nanoparticle concentration is another critical factor. Optimal concentrations maximize charge trapping and PDIV improvements, while excessive concentrations can lead to particle agglomeration and reduced effectiveness. For example, CQD-$SiO_{2}$ nanostructures achieve optimal performance when clustering is avoided [108]. In sunflower oil-based nanofluids, a 0.05% mass concentration of $SiO_{2}$ reduces PD amplitude and pulse duration, enhancing PDIV [111]. Similarly, ferrofluids containing iron oxide nanoparticles perform optimally when their concentration balances charge trapping with fluid stability [112]. Jin et al. [113] emphasized that low nanoparticle concentrations (∼0.01 wt.%) are sufficient to achieve significant PDIV improvements, as seen with $SiO_{2}$ enhancing PDIV by 20% and $C_{60}$ (fullerene) by 10%.

The physical characteristics of nanoparticles—including size, surface area, and shape—also influence their effectiveness in enhancing PDIV. Nanoparticles with a 10 to 50 nm size range and a high surface area provide numerous charge-trapping sites, improving dielectric properties. However, excessively small particles can agglomerate, limiting their effectiveness [114,115]. Conversely, larger or irregularly shaped nanoparticles may concentrate electric fields and reduce breakdown voltage, emphasizing the importance of maintaining proper particle geometry [116].

High bandgap energy is another crucial property directly enhancing PDIV by suppressing charge carrier generation. Materials like $SiO_{2}$, with their large bandgap, are highly effective in reducing free charge carriers, thus minimizing the occurrence of partial discharges [108]. This property complements the charge-trapping ability of nanoparticles and has been shown to improve PDIV significantly. For example, incorporating $SiO_{2}$ nanoparticles in transformer oils enhances charge suppression and improves the insulation’s electrical stability under various operating conditions [111]. Combining high bandgap energy and proper nanoparticle dispersion contributes to more robust insulation performance.

Low conductivity and high breakdown strength are essential properties that prevent unintended charge conduction paths, further enhancing PDIV. Non-conductive nanoparticles, such as hexagonal boron nitride (h-BN), effectively increase dielectric strength by limiting charge conduction within the insulation. Including h-BN and other non-conductive nanoparticles enhances the dielectric performance of the base fluid by preventing localized breakdown events and improving its overall resistance to PD [117]. Studies have shown that including nanoparticles in transformer oils leads to an average improvement of around 21% in breakdown voltage [118]. The dielectric strength of oils has been significantly improved through the addition of various nanoparticles, including $Al_{2}O_{3}$, $SiO_{2}$, and h-BN, which enhance the electrical stability of insulating fluids [119]. Additionally, nano magnesium oxide (*MgO*) and h-BN have shown excellent performance in enhancing the dielectric characteristics of mineral and ester oils, making them ideal for sustainable transformer applications [115,117].

High electron trap density is a crucial property that enhances PDIV. Nanoparticles such as $SiO_{2}$ and $TiO_{2}$ possess deep electron traps that capture free charges, thereby delaying the onset of partial discharges. Silicon nanoparticles form an electrical double layer that further enhances charge trapping [120]. Studies show that minimizing free electron energy reduces PD initiation [109], making this a key mechanism for PDIV improvement.

Dielectric permittivity also plays a significant role in PDIV enhancement by improving charge distribution and reducing local electric field stress. Nanoparticles like $CaCu_{3}Ti_{4}O_{12}$ (CCTO) in synthetic ester oil increase breakdown voltage and reduce the loss tangent, resulting in higher PDIV [121]. Similarly, sunflower oil-based nanofluids containing $SiO_{2}$ exhibit significant improvements due to enhanced polarization and charge-trapping [111]. However, excessive nanoparticle concentrations can lead to the formation of nanosized pores, which facilitate PD activity, underscoring the importance of optimal permittivity control [120].

The stability and uniform dispersion of nanoparticles are essential for ensuring consistent long-term PDIV improvements. Proper dispersion prevents agglomeration, which can cause localized electric field intensification and weaken insulation performance. Hydrophilic nanoparticles, such as $SiO_{2}$, help stabilize the fluid by absorbing moisture and reducing weak points [113]. Dispersants, such as oleic acid, have also been shown to maintain dispersion, ensuring that dielectric improvements are consistent [120]. Without proper stabilization, agglomeration reduces the effectiveness of the nanofluids, leading to decreased insulation performance [122].

In conclusion, optimizing PDIV in transformer insulation involves carefully selecting nanoparticle type, concentration, size, conductivity, and dispersion. Research confirms that $SiO_{2}$, $TiO_{2}$, $Al_{2}O_{3}$, $C_{60}$, h-BN, and SiC nanoparticles contribute to PDIV improvements through mechanisms such as moisture absorption, charge trapping, bandgap energy, and prevention of charge conduction. Further investigation into eco-friendly alternatives and long-term stability will ensure the development of sustainable, high-performance nanofluids for electrical applications.

#### 5.1.3. Viscosity

Viscosity significantly influences insulating fluid’s convective heat transfer efficiency and should ideally be minimized. While some discrepancies exist in the literature, most studies agree that nanofluids exhibit higher viscosity than their base oils due to the increased density from nanoparticle addition [100]. This increase in viscosity could potentially hinder heat transfer, but it is counterbalanced by the enhanced thermal conductivity imparted by nanoparticles [123]. Both base oils and nanofluids show a decline in viscosity with rising temperatures, maintaining similar trends across studies [124].

Surfactants play a critical role in reducing nanofluid viscosity by weakening interparticle bonds and lowering shear resistance. This separation effect increases the total nanoparticle surface area, which enhances the fluid’s thermal conductivity [125,126].

Synthetic esters, known for their higher viscosity than mineral oils, experience a more pronounced absolute increase in viscosity with nanoparticle addition. However, this increase is comparatively smaller when evaluated proportionally to mineral oil-based nanofluids. Similar behaviour is observed in natural esters with inherently high viscosities, as shown in Table 6. The data also highlight the direct relationship between nanoparticle concentration in the base fluid and the overall viscosity.

Regarding viscosity, the trends concerning size are more predictable. The assumption mentioned earlier is supported by the experimental results found in the literature. The vast majority of particles’ incorporation leads to more viscous fluids, regardless of the base fluid. The table also proves that larger particles give rise to more viscous fluids. This trend was observed consistently across most studies, albeit with varying degrees of significance, with one notable exception. Incorporating MWCNT into mineral oil, with particles sizes ranging between 10 and 20 nanometres and a concentration of 0.0001 wt.%, produced nanofluid less viscous than the original mineral oil [106]. According to the authors, this anomaly can be attributed to the lubricating properties of carbon particles which, when combined with a low particle concentration, can reduce viscosity.

The multiplicity of variables in each analysis and the absence of a study comparing the various base fluids for the same conditions and particles do not enable the withdrawal of a comprehensive conclusion in what concerns viscosity variation in absolute terms.

#### 5.1.4. Thermal Conductivity

Studies have demonstrated that nanofluids generally offer better thermal conductivity than pure oils, primarily due to the high conductivity of metallic nanoparticles. While adding nanoparticles enhances thermal conductivity, this improvement does not always lead to a proportional enhancement in heat transfer efficiency, especially at hot spots [132]. For instance, while nanofluids exhibit higher thermal conductivity, the temperature difference at steady-state conditions in transformers may only reduce by a few degrees. However, even a small temperature reduction is significant, as an increase of just a few degrees can halve the transformer’s service life [133]. At low nanoparticle concentrations, the thermal conductivity increases linearly with temperature. However, as the concentration increases, deviations emerge due to nanoparticle agglomeration, which reduces the effective surface area available for heat transfer and diminishes heat transfer efficiency [134]. The use of surfactants can help mitigate agglomeration and maintain more stable thermal conductivity over multiple cycles. Additionally, smaller nanoparticles tend to provide better thermal conductivity than larger ones because they offer a larger surface area at the same volume concentration [135]. Furthermore, rod-shaped particles have been found to be more effective than spherical particles in enhancing thermal conductivity. However, it is important to consider the aspect ratio of the particles, as it can affect the stability of the nanofluid [136]. While synthetic and natural esters exhibit higher thermal conductivity than mineral oils, the overall heat dissipation ability of ester-based nanofluids does not necessarily surpass that of mineral oils, as viscosity plays a crucial role in heat transfer efficiency. As reported in various studies, the thermal conductivity improvements of nanofluids are summarized in Table 7, which details percentage enhancements for different nanoparticle types and concentrations. Moreover, from the studies analysed, it becomes clear that an optimal nanoparticle concentration combined with surface-functionalized nanoparticles offers the best thermal conductivity retention under thermal cycling.

### 5.2. Fluid Preparation, Stability and Ageing

**Figure 6:**
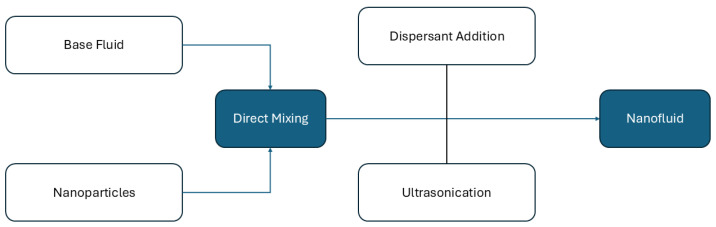
Two-step method for nanofluids preparation.

Ensuring the stability of nanodielectric fluids (NDFs) remains a key challenge in their widespread adoption as commercial transformer liquids. Achieving long-term stability is essential for improving their thermal and dielectric properties [142]. The stability of nanofluids is predominantly influenced by the nanoparticles (NPs) and the base fluids utilized. A prevalent indicator of stability is the aggregation of nanoparticles, which is affected by the thermodynamic attributes of the system and the overall interactions among particles. Due to Brownian motion, nanoparticles smaller than 1 nm frequently collide, leading to the formation of secondary clusters driven by attractive forces. As these clusters grow, aggregation occurs, ultimately causing sedimentation and instability [143]. Controlling the high surface activity and chemical reactivity of nanoparticles is critical for maintaining stability. The key forces affecting NP stability include van der Waals, electrostatic, and gravitational forces, with the former being particularly consequential [144]. The diminutive size of NPs amplifies van der Waals forces, as this force exhibits an inverse relationship with particle diameter [144]. According to the DLVO theory formulated by Derjaguin, Landau, Verwey, and Overbeek, stability is attained through a delicate equilibrium between van der Waals attraction and electrostatic repulsion [145]. When this repulsion is strong enough, nanoparticles remain well-dispersed in the fluid. Nanofluid synthesis generally follows one of two approaches: the one-step method [146] or the two-step method [115]. The two-step process is commonly employed in experimental studies and is illustrated in Figure 6.

**Figure 7:**
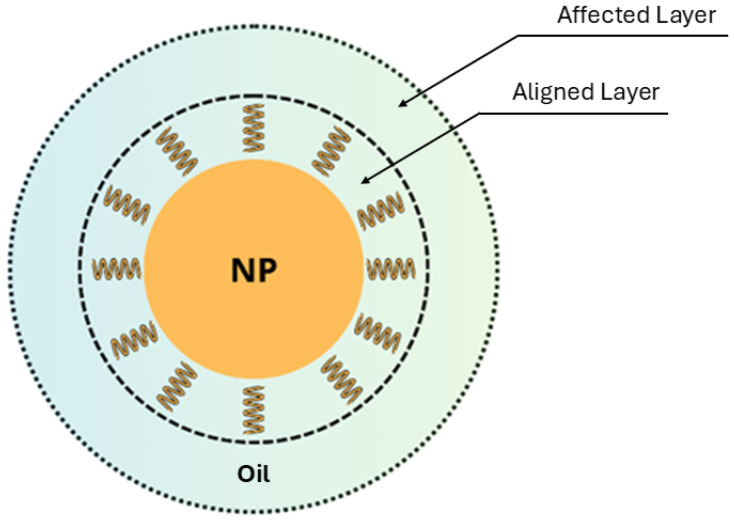
Nanoparticle surfactant layer.

Physical and chemical techniques are often used in what concerns stability improvement. Physical methods primarily focus on reducing particle size using ultrasonic treatment and mechanical stirring. On the other hand, chemical approaches involve adjusting the fluid’s pH and incorporating surfactants to modify nanoparticle surfaces [115]. These modifications alter surface interactions and help prevent aggregation by disrupting attractive forces. Since surfactants facilitate nanoparticle dispersion through different mechanisms, it is essential to consider factors such as the base fluid type, surfactant selection, and concentration when developing stable nanofluids. Commonly used surfactants include oleic acid, cetrimonium bromide (CTAB), sorbitan monooleate (Span^®^ 80), and stearic acid [24]. The addition of surfactants ensures uniform nanoparticle dispersion in the base fluid while also preventing particle agglomeration. Moreover, surfactants form a protective layer around nanoparticles, modifying their surface characteristics. This layer, depicted in Figure 7, helps dissipate energy from mobile charge carriers, thereby delaying electrical breakdown.

To further illustrate the impact of nanoparticle size, concentration, and base fluid composition on nanofluid stability, Table 8 presents a summary of relevant studies from literature. This table highlights the key parameters, including nanoparticle type, concentration, particle size, base fluid, and the reported shelf life of the prepared nanofluids.

Nanoparticle sedimentation has been found to degrade heat transfer efficiency in multi-channel heat sinks (MCHS) [161]. To enhance the stability of nanofluids, surfactants are commonly added [162]. These surfactants adsorb onto the surface of nanoparticles, increasing the interparticle distance and reducing van der Waals interactions by lowering the Hamaker constant. However, the addition of surfactants can alter the properties of nanofluids at elevated temperatures, which affects their applicability in heat exchangers. The bond between surfactants and nanoparticles breaks down at high temperatures, compromising nanofluid stability. Additionally, surfactants such as CTAB generate foam during heating and cooling cycles, reducing thermal conductivity and heat transfer efficiency [163]. The thermal degradation of surfactants further deteriorates nanofluid stability at elevated temperatures [164].

As temperature increases, the zeta potential of nanofluids declines—from 21.7 to 13.4—due to nanoparticle agglomeration and growth in particle size [165]. Additionally, surfactant residues can accumulate on heat exchanger surfaces, further limiting the effectiveness of nanofluids in thermal management applications [166].

Functionalized surface modification of nanoparticles offers a long-term stability solution for nanofluids without requiring surfactants. This modification, achieved through physical and chemical methods, alters surface properties such as atomic layer structure, functional groups, and hydrophobicity. Surface modification enhances nanoparticle dispersion, surface activity, and biocompatibility compared to other stabilisation techniques.

For instance, $SiO_{2}$ nanoparticles are modified using a silane coupling agent (KH570) to improve dispersion stability, achieving a stabilization time of 120 min at 5% KH570 content [167]. Similarly, $SiO_{2}$ nanoparticles are used to modify $Si_{3}N_{4}$ particles to prevent oxidation at high temperatures, forming a uniform $SiO_{2}$ adsorption layer. SEM images confirm that increasing $SiO_{2}$ content results in rougher $Si_{3}N_{4}$ surfaces due to nanoparticle aggregation.

Additionally, surface-modified zero-valent iron nanoparticles exhibit improved stability, fluidity, and reduced environmental toxicity. The hydrophobicity of modified $SiO_{2}$ nanoparticles is enhanced, improving their compatibility with organic environments and significantly increasing the stability of nano-$SiO_{2}$ fluids [168].

The aggregation of nanoparticles in the base fluid significantly influences both thermal conductivity and viscosity [161]. Nanofluids exhibit superior heat transfer performance compared to conventional liquids due to the substantial increase in thermal conductivity provided by the nanoparticles. Key factors affecting the transport properties of nanofluids include nanoparticle volume fraction, material properties, and particle size. Suspension stability plays a crucial role in determining the thermal conductivity of nanofluids. Generally, nanofluids with higher suspension stability exhibit enhanced thermal conductivity. Nanoparticles facilitate energy transfer within the mixture, improving thermal conductivity under cyclic loading. Due to their small size, suspended nanoparticles undergo random motion driven by Brownian forces. This micro-convection effect enhances energy transfer between the particles and the surrounding liquid, increasing the nanofluid’s thermal conductivity. While nanoparticle aggregation can initially enhance thermal conductivity, a critical threshold exists beyond which further aggregation negatively impacts heat transfer efficiency [162]. Over time, thermal conductivity may increase as heat conduction becomes more effective through nanoparticle aggregates. However, prolonged aggregation leads to coalescence and clustering, eventually causing sedimentation due to gravity. This results in a decline in thermal conductivity. To mitigate this effect, it is recommended to control the polymerization rate through chemical or physical stabilization methods [163]. While nanoparticles such as $Al_{2}O_{3}$, $TiO_{2}$, and h-BN initially improve thermal conductivity, sedimentation and clustering over time may reduce these benefits by 10–20% [163].

Research indicates that nanofluids generally perform better than pure liquids in AC-BDV and LI-BDV tests, both in their new and aged states [169,170]. Although ageing processes reduce BDV values for both fluid types, studies suggest that nanofluids experience a slower decline in AC-BDV values [171]. Furthermore, in PDIV tests, nanofluids consistently outperformed pure oils in both new and aged samples [172].

A thorough investigation into the ageing characteristics of each oil referenced in the preceding paragraph, when enhanced with nanoparticles, would require an extensive series of tests, which, to date, appear absent from the existing literature. However, in transformer applications, the ageing status can also be assessed through the condition of insulation paper. While limited, some studies suggest that nanoparticles influence the properties of cellulosic materials. Insulation paper’s physical condition is typically evaluated using Degree of Polymerization (DP) or Tensile Strength (TS) tests [173]. Over a 56-day ageing test, nanofluid-impregnated paper exhibited a 29.4% higher DP value than paper impregnated with mineral oil [174], indicating greater resistance to ageing. Similarly, nanoparticles were found to fill voids in the paper’s structure, forming hydrogen bonds that enhance mechanical strength and thermal stability [175].

Despite these promising findings, gaps remain in understanding the effects of different base fluids on insulation paper, particularly ester-based nanofluids. While DP tests for ester-based nanofluids suggest negative results, it remains unclear whether this outcome is due to the base fluid or the nanoparticles. More comparative research is required to clarify these uncertainties and optimize nanofluid formulations.

## 6. Challenges and Opportunities for Future Research

Nanofluids in transformers face several challenges, including nanoparticle agglomeration and sedimentation, which can degrade insulation and increase the risk of raising electrical failure risk. Stability is crucial, as disruptions negate the intended benefits of nanofluids. High nanoparticle concentrations can reduce effectiveness, emphasizing size and concentration optimization. Furthermore, traditional regeneration systems are incompatible with nanofluids, leading to increased maintenance and accelerated ageing costs.

**Figure 8:**
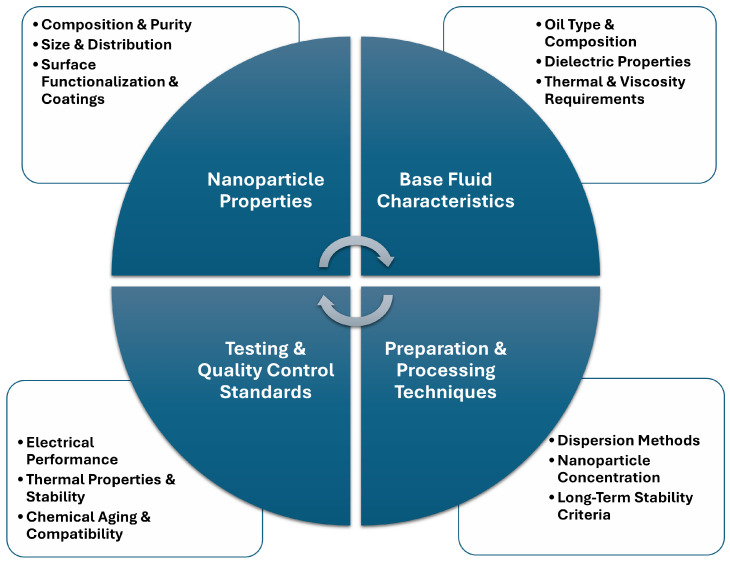
Roadmap for nanofluid-based transformer insulation scalability.

Insulating nanofluids are more expensive than pure insulating fluids, and scaling up production for transformer applications can result in longer fabrication times. Additionally, nanoparticles’ potential corrosive and toxic effects must be carefully managed during production to ensure safety and reliability. Production methodologies also present significant obstacles. While offering enhanced stability, the one-step synthesis approach has scalability limitation, rendering it unsuitable for large-scale manufacturing. In contrast, the two-step method, more feasible for industrial applications, often compromises nanofluid stability due to nanoparticle aggregation during storage and transport. Stabilisation techniques such as surfactant modification, ultrasonic dispersion and pH control enhance compatibility between nanoparticles and base fluids. However, excessive use of stabilisers may adversely affect the dielectric properties, further complicating the optimisation process. It is also fundamental to establish uniform testing protocols to validate: electrical performance—Dielectric breakdown (IEC 60156), PDIV (IEC 60270 [176]), and space charge analysis (PEA method); thermal properties & stability—thermal conductivity (ISO 22007-2 [177]), viscosity (ASTM D445 [178]), flash point, and oxidation stability (IEC 61125 [179]); chemical ageing & compatibility performance. Economic and environmental considerations add another layer of complexity to nanofluid adoption. Manufacturing costs remain high due to the complexity of synthesis and the necessity of specialised additives. Furthermore, specific nanoparticles’ inherent toxicity and potential adverse interactions with other transformer components raise significant safety and environmental concerns. Research efforts increasingly focus on hybrid nanoparticle systems to harness synergistic effects on thermal and electrical characteristics while mitigating production challenges. Figure 8 summarises the key parameters to ensure consistent performance, reproducibility, and scalable production of these nanofluids. The standardization of the highlighted parameters across these four detrimental categories (i.e nanoparticle properties, base fluid characteristics, preparation and processing techniques, testing and quality control standards), minimises variability, enhances reproducibility, and will enable scalable production of nanofluid-based insulating liquids.

Based on the comprehensive analysis presented in this review, the authors propose that while nanofluids hold significant potential, their practical application faces substantial challenges demanding a multidisciplinary evaluation. This evaluation should prioritize identifying the precise thermal, dielectric, and rheological properties required for nanofluids to meet the operational and reliability standards of transformer systems. Additionally, the authors recommend that Computational Fluid Dynamics (CFD) modelling and simulation play a central role in these effort, providing a robust framework to investigate heat transfer dynamics, fluid flow behaviour, and electric field interactions under varied operational conditions. When combined with experimental validation, CFD-driven methodologies are anticipated to facilitate the required systematic pathway to attain scalable production of nanofluid based insulating liquids.

## 7. Conclusions

This review has holistically examined the advancements and challenges in the application of dielectric nanofluids for power transformer cooling. By evaluating the electrical, physical, and thermochemical properties of these nanofluids, the study has provided a comparative assessment of the applied base fluids, nanoparticle types, nanofluid preparation techniques, mixing ratios, stabilisation methods, and corresponding results. The findings reveal that nanofluids offer significant advantages over traditional insulating fluids, particularly in enhancing dielectric properties, cooling efficiency, and overall performance.

The integration of nanoparticles into insulating fluids has demonstrated a substantial improvement in BDV, even at elevated temperatures, with recorded improvements ranging from 1% to 202%, depending on nanoparticle type, concentration, and dispersion techniques. This improvement is attributed to the ability of nanoparticles to slow streamer propagation and prolong breakdown time, provided that nanoparticle concentrations are optimised to prevent agglomeration and adverse effects. The higher partial discharge inception voltage observed in nanofluids further highlights their ability to delay and mitigate partial discharge phenomena compared to conventional insulating oils. Specifically, TiO_2_ and ZnO nanoparticles have demonstrated the highest performance, with optimal BDV increases occurring at concentrations below 0.3 g/L for particles smaller than 50 nm. Additionally, PDIV improvements of up to 23% have been reported for carbon quantum dot-coated silica nanoparticles, which exhibit superior electron-trapping properties. However, the dielectric performance of nanoparticles can vary based on the properties of the base fluid, with mineral oil-based nanofluids generally outperforming their vegetable-based counterparts.

Thermally, the suspension of nanoparticles improves heat dissipation due to enhanced thermal conductivity, with increases of 5–20%, offering a distinct advantage in mitigating hotspots within oil-immersed transformers. While an increase in viscosity, ranging from 3–21%, is noted with nanoparticle addition, this can be offset by temperature increases and the use of surfactants, which improve fluid flow without compromising thermal performance. The higher flash points observed in nanofluids also enhance their safety in high-temperature applications. Importantly, the positive effects of nanoparticles extend to aged or moisture-containing fluids, where they have been shown to improve dielectric properties and slow the degradation of cellulosic insulation materials.

Despite these promising findings, significant variability exists in reported results due to differences in nanoparticle size, shape, concentration, stabilisation techniques, and testing methodologies. This variability underscores the necessity for standardised processes in nanofluid preparation and evaluation. Establishing well-defined testing protocols and conducting long-term studies under field conditions will be critical to understanding the ageing behaviour, stability under magnetic fields, cooling performance, and electrical properties of nanofluids over time.

In conclusion, while the theoretical and experimental advancements in dielectric nanofluids offer compelling prospects for improving transformer performance, their practical implementation will require a multidisciplinary approach. Future research should focus on multivariate analyses to identify optimal formulations, comprehensive field testing to validate long-term reliability, and the development of scalable, cost-effective production techniques. By addressing these challenges, the electricity industry can harness the full potential of nanofluids, advancing towards more efficient, reliable, and sustainable transformer technologies.

## Figures and Tables

**Table 1:** Properties of different types of esters, mineral and silicone oils [49,50,51].

Property	Mineral Oil	Natural Ester				Synthetic Ester	Silicone Oil
		Sunflower	Rapeseed	Soybean	Palm		
Density at 20 °C [g·cm^−3^]	0.839	0.91	0.92	0.92	0.97	0.839	0.96–1.10
Kinematic viscosity at 40 °C [mm^2^·s^−1^]	9.98	39.2	37	34	5.062	29	39
Flash point [°C]	176	330	>315	320	188	260	300
Fire point [°C]	-	362	>350	350	206	316	-
Pour point [°C]	−48	−25	−31	−18	−37.5	−56	−50
Acidity [mg (KOH)·g^−1^]	<0.01	0.05	≤0.04	<0.05	<0.01	<0.03	-
Moisture [mg (H_2_O)·kg^−1^]	15	150	50	4–50	52	50	80
Dielectric dissipation factor at 90 °C	0.002	0.03	<0.03	<0.03	<0.003	<0.008	-
Breakdown voltage [kV]	46	65	>75	≥55	85	>75	40
Biodegradability [%]	-	85	98	>99	77	89	-

**Table 2:** Properties Standard Tests.

Property	Standard Test
Breakdown voltage [kV]	IEC 60156 [60]
Dielectric dissipation factor at 90 °C	IEC 60247 [61]
Moisture content [ppm]	IEC 60814 [62]
Acidity [mg·KOH/g]	IEC 62021 [63]
Flash Point [°C]	ASTM D92 [64]
Fire Point [°C]	ASTM D93 [65]

**Table 3:** Modelling of temperature dependent properties.

Fluid	Property	Equation
	Density [g·cm^−3^]	ρ=1098.72−0.712·T
Mineral Oil	Conductivity [W·m^−1^·K^−1^]	$k=0.1509-7.101\;\cdot \;10^{-5}\;\cdot \;T$
[66]	Specific Heat [J·kg^−1^·K]	$c_{p}=807.163+3.58\;\cdot \;T$
	Viscosity [mm^2^·s^−1^]	$\mu =0.08467-0.0004\;\cdot \;T+5\;\cdot \;10^{-7}\;\cdot \;T^{2}$
	Density [g·cm^−3^]	ρ=1185−0.7333·T
Synthetic	Conductivity [W·m^−1^·K^−1^]	$k=9.71\;\cdot \;10^{-2}+3.74\;\cdot \;10^{-4}\;\cdot \;T-7.2503\;\cdot \;10^{-7}\;\cdot \;T^{2}$
Ester Oil	Specific Heat [J·kg^−1^·K]	$c_{p}=1242.4+2.198\;\cdot \;T$
[67]	Viscosity [mm^2^·s^−1^]	$\mu =0.2565-1.2963\;\cdot \;10^{-3}\;\cdot \;T+1.6761\;\cdot \;10^{-6}\;\cdot \;T^{2}$
	Density [g·cm^−3^]	ρ=1118.1−0.6737·T
Natural	Conductivity [W·m^−1^·K^−1^]	$k=0.2241-2.1174\;\cdot \;10^{-4}\;\cdot \;T$
Ester Oil	Specific Heat [J·kg^−1^·K]	$c_{p}=986.78+3.9658\;\cdot \;T$
[29]	Viscosity [mm^2^·s^−1^]	$\mu =1.19\;\cdot \;10^{-4}\;\cdot \;e^{\left(\frac{924}{T-147}\right)}$

**Table 4:** Properties of the most common nanoparticles [23,37,71].

Nanoparticle	*Fe*_3_ *O*_4_	*Al*_2_ *O*_3_	*TiO*_2_	*SiO*_2_	*ZnO*
Density [g·cm^−3^]	5.2	3.9	4.2–4.3	2.2	5.6
Relative permittivity	80.0	9.9	3.0–114.0	3.8	7.4–8.9
Relaxation time [s]	7.5 × 10^−14^	42.5	3.1 × 10^−11^	5.1 × 10^−2^	1.1 × 10^−11^
Electric Conductivity [S·m^−1^]	10.0^4^	1.0 × 10^−10^	1.0 × 10^−12^	1.4 × 10^−9^	10.0–10.0^3^
Thermal Conductivity [W·m^−1^·K^−1^]	4.0–8.0	30.0	-	1.4	23.4
Thermal Expansion Coefficient at 20 °C [µm·m^−1^·K^−1^]	9.2	-	-	30.0	2.9
Specific Heat [J·kg^−1^·K^−1^]	-	850	-	670.0	494.0

**Table 5:** Base and Nanofluids BDV Comparison.

Base Fluid (BF)	NP	NP Size [nm]	NP Concentration	Preparation Method	Gap Distance [mm]	BF BDV [kV]	NDF BDV [kV]	BDV Improvement [kV]	Source
Natural Ester	$TiO_{2}$	21	0.02 vol%	two-step	2.5	68	71	22.4	[84]
Natural Ester	h-BN	50–70	0.1 wt.%	two-step	2.5	37.6	61.4	63.3	[85]
Mineral Oil	$TiO_{2}$	60–70	0.025 wt.%	one-step	2	52.5	72.6	38.3	[86]
Mineral Oil	$Al_{2}O_{3}$	60–70	0.025 wt.%	one-step	2	52.5	66.2	26.09	[86]
Mineral Oil	$MoS_{2}$	60–70	0.025 wt.%	one-step	2	52.5	59.2	12.8	[86]
Mineral Oil	$TiO_{2}$	10–20	0.05 g/L	two-step	–	15.65	21.32	36.23	[87]
Mineral Oil	$SiO_{2}$	25	0.2 g/L	two-step	2	12	29.88	149.0	[88]
Mineral Oil	$SiO_{2}$	25	0.2 g/L	two-step	2	9	14.58	62.0	[88]
Mineral Oil	$TiO_{2}$	25	0.2 g/L	two-step	2	12	37.68	214.0	[88]
Mineral Oil	$TiO_{2}$	25	0.2 g/L	two-step	2	9	24.21	169.0	[88]
Mineral Oil	$ZrO_{2}$	25	0.2 g/L	two-step	2	12	32.88	174.0	[88]
Mineral Oil	$ZrO_{2}$	25	0.2 g/L	two-step	2	9	21.06	134.0	[88]
Natural Ester	$Fe_{2}O_{3}$	10–20	0.3 g/L	two-step	–	35	38	8.00	[89]
Mineral Oil	$Al_{2}O_{3}$	20	0.05 g/L	two-step	2.5	51.18	60.6	21.2	[90]
Mineral Oil	$TiO_{2}$	20	0.05 g/L	two-step	2.5	51.18	57.3	14.6	[90]
Mineral Oil	ZnO	20	0.05 g/L	two-step	2.5	51.18	67.3	34.6	[90]
Natural Ester	$Fe_{3}O_{4}$	50	0.4 g/L	–	2.5	68.77	73.63	7.07	[83]
Natural Ester	$Fe_{3}O_{4}$	50	0.3 g/L	–	2.5	68.77	69.53	1.1	[83]
Natural Ester	$Al_{2}O_{3}$	13	0.05 g/L	–	2.5	68.77	73.93	7.5	[83]
Natural Ester	$Al_{2}O_{3}$	13	0.3 g/L	–	2.5	68.77	67.33	−2.09	[83]
Natural Ester	$Al_{2}O_{3}$	50	0.3 g/L	–	2.5	68.77	73	6.15	[83]
Natural Ester	$SiO_{2}$	10–20	0.3 g/L	–	2.5	68.77	72.73	5.76	[83]
Synthetic Ester	$Fe_{3}O_{4}$	50	0.4 g/L	two-step	2.5	60	88.67	47.8	[91]
Synthetic Ester	$Al_{2}O_{3}$	13	0.05 g/L	two-step	2.5	60	80.83	34.72	[91]
Synthetic Ester	$Al_{2}O_{3}$	13	0.4 g/L	two-step	2.5	60	69.9	16.5	[91]
Synthetic Ester	$Al_{2}O_{3}$	50	0.3 g/L	two-step	2.5	60	75.27	25.45	[91]
Synthetic Ester	$Al_{2}O_{3}$	50	0.4 g/L	two-step	2.5	60	74.03	23.38	[91]
Synthetic Ester	$SiO_{2}$	10–20	0.4 g/L	two-step	2.5	60	78.9	31.5	[91]
Mineral Oil	$TiO_{2}$	46	0.05 vol%	–	2	71	79	11.27	[92]
Mineral Oil	$TiO_{2}$	105	0.05 vol%	–	2	71	89	25.35	[92]
Mineral Oil	ZnO	34	0.05 vol%	–	2	71	77	8.45	[92]
Mineral Oil	$TiO_{2}$	5	0.075 vol%	–	2	30	59	96.7	[93]
Mineral Oil	$TiO_{2}$	10	0.075 vol%	–	2	30	63	110.0	[93]
Mineral Oil	$TiO_{2}$	15	0.075 vol%	–	2	30	32	6.67	[93]
Mineral Oil	$TiO_{2}$	100	0.3 g/L	two-step	2.7	12	26	116.7	[94]
Mineral Oil	$Fe_{3}O_{4}$	–	0.4 g/L	–	2	60	70.5	17.5	[95]
Mineral Oil	$TiO_{2}$	100	0.06 g/L	two-step	1.5	23.6	29.97	27.0	[96]
Mineral Oil	$Al_{2}O_{3}$	13	0.05 g/L	two-step	2.5	38.5	67.9	76.36	[97]
Mineral Oil	$Al_{2}O_{3}$	13	0.3 g/L	two-step	2.5	38.5	58.1	50.1	[97]
Mineral Oil	$Al_{2}O_{3}$	50	0.3 g/L	two-step	2.5	38.5	65.1	69.1	[97]
Mineral Oil	$Al_{2}O_{3}$	50	0.05 g/L	two-step	2.5	38.5	52.7	36.89	[97]
Mineral Oil	$SiO_{2}$	10–20 mn	0.01 wt.%	two-step	2.5	30.39	41.28	35.83	[98]
Mineral Oil	$TiO_{2}$	17.6	0.075 Φ%	two-step	–	60	79	31.67	[99]
Mineral Oil	ZnO	>100	0.03 Φ%	two-step	2.5	50.5	72.1	42.77	[100]
Mineral Oil	ZnO	>100	0.03 Φ%	two-step	2.5	54.3	76.2	40.33	[100]
Mineral Oil	ZnO	>100	0.03 Φ%	two-step	2.5	62.4	78.6	25.96	[100]
Mineral Oil	$TiO_{2}$	>100	0.03 Φ%	two-step	2.5	50.5	64.3	28.0	[100]
Mineral Oil	$TiO_{2}$	>100	0.03 Φ%	two-step	2.5	54.3	72.2	35.0	[100]
Mineral Oil	$TiO_{2}$	>100	0.03 Φ%	two-step	2.5	62.4	74.6	19.0	[100]
Mineral Oil	$BaTiO_{3}$	>100	0.03 Φ%	two-step	2.5	50.5	69.7	38.0	[100]
Mineral Oil	$BaTiO_{3}$	>100	0.03 Φ%	two-step	2.5	54.3	74.8	37.0	[100]
Mineral Oil	$BaTiO_{3}$	>100	0.03 Φ%	two-step	2.5	62.4	77.3	24.0	[100]
Mineral Oil	$Fe_{2}NiO_{4}$	50	0.06 g/L	two-step	2	18.475	22.96	24.27	[101]
Mineral Oil	CdS	4.5	0.06 g/L	two-step	2	18.475	19.225	4.06	[101]
Mineral Oil	$SiO_{2}$	–	0.2 vol%	one-step	2	60.9	72.9	19.7	[102]
Mineral Oil	$TiO_{2}$	>20	0.006 g/L	one-step	2.5	71.59	82.48	15.21	[103]
Natural Ester	$CCl_{4}$	–	0.5 g/L	–	2.5	68.77	79.5	15.6	[104]
Natural Ester	$CCl_{4}$	–	0.4 g/L	–	2.5	68.77	68.4	−1.00	[104]
Natural Ester	$CH_{3}I$	–	0.5 g/L	–	2.5	68.77	66.4	−3.45	[104]
Natural Ester	$CH_{3}I$	–	0.4 g/L	–	2.5	68.77	70.3	2.20	[104]
Mineral Oil	$Fe_{3}O_{4}$	30–40	0.2 mg/L	two-step	2.5	42.6	57.3	34.00	[105]
Mineral Oil	$Fe_{3}O_{4}$	50–100	0.02 g/L	two-step	–	42.141	53.841	27.76	[77]
Mineral Oil	$TiO_{2}$	80–120	0.02 g/L	two-step	–	42.141	50.733	20.39	[77]
Synthetic Ester	BCZT	50–100	0.005 wt.%	two-step	–	60	72	20.00	[77]
Natural Ester	SiC	50	0.004 wt.%	two-step	2.5	61.3	71.3	16.31	[106]
Natural Ester	$Al_{2}O_{3}$	50	0.004 wt.%	two-step	2.5	61.3	63.8	4.08	[106]
Mineral Oil	$ZrO_{2}$	30	0.001 wt.%	two-step	2	11.39	34.47	202.6	[107]

**Table 6:** Viscosity comparison of base fluids and nanofluids.

BF	NP	NP Size [mm]	NP Concentration	Temperature [°C]	BF Viscosity	NDF Viscosity	Viscosity Rise [%]	Source
Mineral Oil	AlN	40	0.1 wt.%	60	3.75 mm_2_/s	4 mm_2_/s	6.67	[127]
Mineral Oil	Al_2_O_3_	20	0.05 g/L	–	11.8 mm^2^/s	12.2 mm_2_/s	3.39	[90]
Mineral Oil	ZnO	20	0.05 g/L	–	11.8 mm^2^/s	11.9 mm^2^/s	0.85	[90]
Mineral Oil	SiO_2_	10–20	0.01 wt.%	25	11.5 mm^2^/s	14 mm^2^/s	0.22	[98]
Mineral Oil	SiO_2_	10–20	0.01 wt.%	70	5.8 mm_2_/s	7.5 mm^2^/s	0.29	[98]
Mineral Oil	TiO_2_	17.6	0.075 Φ%	29	12.4 mPas	12.5 mPas	0.01	[99]
Mineral Oil	Al_2_O_3_	20	0.1 Φ%	20	13 mm^2^/s	14.5 mm^2^/s	0.12	[128]
Mineral Oil	MWCNT	10–20	0.001 wt.%	40	8.111 mm^2^/s	7.873 mm^2^/s	−2.69	[129]
Mineral Oil	MWCNT	10–20	0.001 wt.%	80	3.39 mm^2^/s	3.301 mm^2^/s	−2.54	[129]
Natural Ester	Fe_3_O_4_	–	0.1 g/L	–	44 mm^2^/s	44.55 mm^2^/s	0.01	[130]
Natural Ester	Fe_4_O_4_	–	0.2 g/L	–	44 mm^2^/s	44.75 mm^2^/s	1.70	[130]
Natural Ester	Fe_3_O_4_	–	0.4 g/L	–	44 mm^2^/s	44.9 mm^2^/s	2.04	[130]
Natural Ester	TiO_2_	45	0.04 wt.%	20	66 mPas	73.4 mPas	11.2	[131]
Natural Ester	ZnO	60	0.04 wt.%	20	66 mPas	80 mPas	21.3	[131]
Natural Ester	TiO_2_	45	0.04 wt.%	40	37 mPas	40 mPas	8.10	[131]
Natural Ester	ZnO	60	0.04 wt.%	40	37 mPas	40 mPas	8.10	[131]

**Table 7:** Thermal conductivity comparison of based fluids and corresponding nanofluids.

Base Fluid	NP	NP Size [nm]	NP Concentration	Temperature [°C]	Base Fluid Thermal Conductivity [W·m^−1^·K^−1^]	Nanofluid Thermal Conductivity [W·m^−1^·K^−1^]	Thermal Conductivity Enhancement [%]	Source
Mineral Oil	TiO_2_	17.6	0.075 Φ%	-	0.3714	0.3758	1.2	[137]
Mineral Oil	Al_2_O_3_	20	0.5 Φ%	20	0.116	0.122	5.2	[128]
Mineral Oil	Al_2_O_3_	20	0.5 Φ%	30	0.125	0.135	8.00	[128]
Mineral Oil	MWCNT	10–20	0.01 wt.%	20	0.109	0.11	0.9	[129]
Mineral Oil	MWCNT	10–20	0.01 wt.%	40	0.107	0.111	3.74	[129]
Mineral Oil	MWCNT	10–20	0.01 wt.%	60	0.104	0.112	7.7	[129]
Mineral Oil	SIO_2_	10–20	0.1 wt.%	40	0.1097	0.108	−1.6	[138]
Mineral Oil	AlN	40	0.079 vol%	70	0.0998	0.104	4.2	[138]
Synthetic Ester	CCTO	50–100	0.005 wt.%	RT	0.152	0.198	30.26	[139]
Synthetic Ester	BCZT	50–100	0.005 wt.%	RT	0.152	0.198	30.26	[139]
Synthetic Ester	TiO_2_	21	0.816 g/L	40	0.156	0.16	2.56	[140]
Synthetic Ester	TiO_2_	21	0.816 g/L	80	0.151	0.154	1.99	[140]
Natural Ester	Al_2_O_3_	60	0.04 wt.%	-	0.174	0.185	6.32	[141]

**Table 8:** Overview of literature on the shelf life of power transformer nanofluids.

Base Fluid	Nanoparticle	Size [nm]	Concentration	Shelf Life	Source
Mineral Oil	AlN	-	0.5%	Negligible	[147]
Mineral Oil	SiC|Fe_2_O_3_|SiO_2_	<100	[0.1–5.0] %	Negligible	[148]
Mineral Oil	Al_2_O_3_	<50	[0.1–0.6] g/L	Negligible	[149]
Mineral Oil	Fe_3_O_4_	[20–50]	[0.1–0.6] g/L	1+ day	[150]
Mineral Oil	CuO|SiO_2_|MWCNT	33|12|30	1 vol. %	1+ day	[151]
Mineral Oil	Magnetite	<10	[0.1–0.5] g/L	1+ day	[152]
Synthetic Ester	Fe_3_O_4_	<10	[0.1–0.6] g/L	1+ day	[153]
Mineral Oil	Fe_3_O_4_	<10	0.1 g/L	1 month	[154]
Mineral Oil	TiO_2_	[50–70]	0.005 wt.%	2 months	[155]
Mineral Oil	TiO_2_	[50–70]	0.075 wt.%	2 months	[99]
Synthetic Ester	TiO_2_	∼6	0.075 wt.%	6 months	[99]
Mineral Oil	TiO_2_	<20	[0.005–0.075] wt.%	6 months	[156]
Natural Ester	Fe_3_O_4_|TiO_2_|Al_2_O_3_	[15–20]	0.01 g/L	6 months	[157]
Mineral Oil	C_60_	-	0.15 g/L	12 months	[158]
Mineral Oil	Ceria	[6–8]	[0.1–5.0] wt.%	12 months	[159]
Mineral Oil	Fe_3_O_4_	∼6	[0.28–0.31] g/L	24 months	[160]

## Data Availability

No new data were created or analyzed in this study.

## Abbreviations

The following abbreviations are used in this manuscript:

BDV	Breakdown Voltage
BF	Base Fluid
HMWH	High Molecular Weight Hydrocarbon
MWCNT	Multi-Walled Carbon Nanotubes
NP	Nanoparticle
NDF	Nanodielectric Fluids
PDIV	Partial Discharge Inception Voltage
PT	Power Transformer
RT	Room temperature

## References

[1] Gatto A. (2022). The energy futures we want: A research and policy agenda for energy transitions. Energy Res. Soc. Sci..

[2] Smitkova M., Janicek F., Martins F. Energy Dependency: Worldwide Energy Situation. Proceedings of the 2022 22nd International Scientific Conference on Electric Power Engineering, EPE.

[3] Demarbis A. (2016). Future Energy Systems. Energy Sources Part A Recover. Util. Environ. Eff..

[4] Chu S., Majumdar A. (2012). Opportunities and challenges for a sustainable energy future. Nature.

[5] Holechek J.L., Geli H.M., Sawalhah M.N., Valdez R. (2022). A Global Assessment: Can Renewable Energy Replace Fossil Fuels by 2050?. Sustainability.

[6] Shahidehpour M. (2019). Transportation Electrification: Enhancing the Operation and Control of Electricity and Transportation Infrastructures [About This Issue]. IEEE Electrif. Mag..

[7] Burlig F., Bushnell J., Rapson D., Wolfram C. (2021). The Electricity Grid of the Future—Low Energy: Estimating Electric Vehicle Electricity Use. AEA Pap. Proc..

[8] Revesz R.L., Unel B. (2020). Managing the future of the electricity grid: Modernizing rate design. Harv. Envtl. L. Rev..

[9] Alonzo R.J. (2009). Electrical Codes, Standards, Recommended Practices and Regulations: An Examination of Relevant Safety Considerations.

[10] Koutoua K., Fofana I., Volat C., Farinas M.I. Impact of Oils Degradation on the Cooling Capacity of Power Transformers. Proceedings of the an Unspecified Conference.

[11] Bashi S., Abdullahi U., Yunus R., Nordin A. (2006). Use of natural vegetable oils as alternative dielectric transformer coolants. J.-Inst. Eng..

[12] Fofana I., Benabed F. Influence of Ageing onto the Dielectric Response in Frequency Domain of Oil Impregnated Paper Insulation Used in Power Transformers. Proceedings of the 9ème Conférence Nationale sur la Haute Tension.

[13] Stylianos K. (2009). Distribution Transformers and Maintenance.

[14] Harlow J.H. (2004). Electric Power Transformer Engineering.

[15] Perrier C., Beroual A. (2010). Experimental Investigations on Insulating Liquids for Power Transformers: Mineral, Ester, and Silicone Oils. Electr. Insul. Mag. IEEE.

[16] Rouse T. (1998). Mineral insulating oil in transformers. IEEE Electr. Insul. Mag..

[17] Hiziroglu H. (2010). Conference report: 2009 IEEE Conference on Electrical Insulation and Dielectric Phenomena (CEIDP). IEEE Electr. Insul. Mag..

[18] Aluyor E.O., Ori-Jesu M. (2009). Biodegradation of mineral oils—A review. Afr. J. Biotechnol..

[19] Beroual A., Khaled U., Noah P.S.M., Sitorus H. (2017). Comparative study of breakdown voltage of mineral, synthetic and natural oils and based mineral oil mixtures under AC and DC voltages. Energies.

[20] Azli S.A., Hezri Fazalul Rahiman M., Yusoff Z.M., Fadzilah Razali N., Abd Wahid S.S., Sufian Ramli M. A Review on Alternative Oils as Dielectric Insulating Fluids on Power Transformer. Proceedings of the 2019 IEEE 15th International Colloquium on Signal Processing & Its Applications (CSPA).

[21] Fofana I., Wasserberg V., Borsi H., Gockenbach E. (2002). Challenge of mixed insulating liquids for use in high-voltage transformers.1. Investigation of mixed liquids. Electr. Insul. Mag..

[22] McShane C., Gauger G., Luksich J. Fire resistant natural ester dielectric fluid and novel insulation system for its use. Proceedings of the 1999 IEEE Transmission and Distribution Conference (Cat. No. 99CH36333).

[23] Rafiq M., Lv Y., Li C. (2016). A Review on Properties, Opportunities, and Challenges of Transformer Oil-Based Nanofluids. J. Nanomater..

[24] Farade R.A., Abdul Waha N.I., Mansour D.E.A., Junaidi N., Soudagar M.E.M., Rajamony R.K., AlZubaidi A. (2024). A review on ultrasonic alchemy of oil-based nanofluids for cutting-edge dielectric and heat transfer oils. J. Mol. Liq..

[25] Kalakonda S.P., Ghassemi M. Nanodielectric Fluids for Power Transformer Cooling and Insulation: A Review. Proceedings of the 2024 IEEE 17th Dallas Circuits and Systems Conference (DCAS).

[26] Sorte S., Martins N., Oliveira M.S.A., Vela G.L., Relvas C. (2023). Unlocking the Potential of Wind Turbine Blade Recycling: Assessing Techniques and Metrics for Sustainability. Energies.

[27] Adekunle A., Oparanti S., Fofana I. (2023). Performance Assessment of Cellulose Paper Impregnated in Nanofluid for Power Transformer Insulation Application: A Review. Energies.

[28] Ponmathy M., Sumathi S., Jumagi R., Rajesh R. (2024). A study on electro-thermal-physical properties of mixed mineral oil and vegetable oil as an alternative for liquid insulation in power transformers. Energy Sources Part A Recover. Util. Environ. Eff..

[29] Wang S., Wang G., Dai W., Zhuo R., Peng Q., Gao M., Zou D., Tang Z., Zhang X. Numerical Calculation and Analysis of Temperature Rise in Power Transformers with Different Insulating Liquids. Proceedings of the 2024 IEEE Electrical Insulation Conference (EIC).

[30] Medeiros L., Oliveira M.M., Beltrame R.C., Bender V.C., Marchesan T.B., Marin M.A. Thermal Experimental Evaluation of a Power Transformer Under OD/OF/ON Cooling Conditions. Proceedings of the 2023 15th Seminar on Power Electronics and Control (SEPOC).

[31] Taghikhani M.A. (2024). Oil Immersed Distribution Transformer HST Reduction using Vegetable Oils and ONAN Cooling. J. Appl. Comput. Mech..

[32] Faiz J., Haghdoust V., Samimi M. (2024). Physical, semi-physical and computational fluid dynamics thermal models of power transformers using artificial neural networks—A review. Int. Commun. Heat Mass Transf..

[33] Zhang Q., Han X., Pu J. (2018). In situ chemosynthesis of TiO2 nanoparticles to endow paper with high water-resistance and retention rate properties. Appl. Phys. A.

[34] Emsley A., Xiao X., Heywood R., Ali M. (2000). Degradation of cellulosic insulation in power transformers. Part 3: Effects of oxygen and water on ageing in oil. IEE Proc.-Sci. Meas. Technol..

[35] Zheng H., Yang E., Wu S., Lv W., Yang H., Li X., Luo X., Hu W. (2022). Investigation on Formation Mechanisms of Carbon Oxides During Thermal Aging of Cellulosic Insulating Paper. IEEE Trans. Dielectr. Electr. Insul..

[36] Saha T., Darveniza M., Yao Z., Hill D., Yeung G. (1999). Investigating the effects of oxidation and thermal degradation on electrical and chemical properties of power transformers insulation. IEEE Trans. Power Deliv..

[37] Arifunnisa S., Champa V., Boddapati V. Investigations on the Impact of Nanoparticles on the Performance of Natural Ester Based Dielectric Coolants for Power Transformer. Proceedings of the 2024 6th International Symposium on Advanced Electrical and Communication Technologies (ISAECT).

[38] Darwin A., Perrier C., Foliot P. The use of natural ester fluids in transformers. Proceedings of the MATPOST conference.

[39] Montero A., García D., García B., Burgos J.C. A comparative study on the dielectric properties of mineral oils and natural esters. Proceedings of the 2023 IEEE International Conference on Environment and Electrical Engineering and 2023 IEEE Industrial and Commercial Power Systems Europe (EEEIC/I&CPS Europe).

[40] Oparanti S.O., Rao U.M., Fofana I. (2023). Natural Esters for Green Transformers: Challenges and Keys for Improved Serviceability. Energies.

[41] Rao U.M., Fofana I., Sarathi R. (2021). Alternative Liquid Dielectrics for High Voltage Transformer Insulation Systems: Performance Analysis and Applications.

[42] Hao J., Zhang J., Ye W., Liao R., Yang L. (2024). Development of Mixed Insulation Oil as Alternative Liquid Dielectric: A Review. CSEE J. Power Energy Syst..

[43] Dombek G., Gielniak J. (2018). Fire safety and electrical properties of mixtures of synthetic ester/mineral oil and synthetic ester/natural ester. IEEE Trans. Dielectr. Electr. Insul..

[44] Rapp K., Gauger G., Luksich J. Behavior of ester dielectric fluids near the pour point. Proceedings of the 1999 Annual Report Conference on Electrical Insulation and Dielectric Phenomena (Cat. No. 99CH36319).

[45] Karthik M., Nuvvula R.S., Dhanamjayulu C., Khan B. (2024). Appropriate analysis on properties of various compositions on fluids with and without additives for liquid insulation in power system transformer applications. Sci. Rep..

[46] Zdanowski M. (2020). Electrostatic Charging Tendency Analysis Concerning Retrofilling Power Transformers with Envirotemp FR3 Natural Ester. Energies.

[47] Montero A., García B., Burgos J.C., González-García C. (2022). Dielectric Design of Ester-Filled Power Transformers: AC Stress Analysis. IEEE Trans. Power Deliv..

[48] S G., Raja T S.R. A Comprehensive Survey on Alternating Fluids Used For The Enhancement of Power Transformers. Proceedings of the 2021 IEEE International Conference on the Properties and Applications of Dielectric Materials (ICPADM).

[49] Méndez C., Olmo C., Antolín I., Ortiz A., Renedo C.J. (2024). Analysing the Suitability of Using Different Biodegradable Fluids for Power Transformers with Thermally Upgraded Paper. Sustainability.

[50] Miller R.E. (1981). Silicone Transformer Liquid: Use, Maintenance, and Safety. IEEE Trans. Ind. Appl..

[51] Moraru G.M., Niagu A., Viziteu G., Andrei P., Florean B. Studies about the breakdown voltage of some liquids insulators. Proceedings of the 2012 International Conference and Exposition on Electrical and Power Engineering.

[52] Fernandez I., Ortiz A., Delgado F., Renedo C., Pérez S. (2013). Comparative evaluation of alternative fluids for power transformers. Electr. Power Syst. Res..

[53] Beroual A., Sitorus H.B.H., Setiabudy R., Bismo S. (2018). Comparative study of AC and DC breakdown voltages in Jatropha methyl ester oil, mineral oil, and their mixtures. IEEE Trans. Dielectr. Electr. Insul..

[54] Das A.K. (2023). Comparative analysis of AC breakdown properties of Jatropha-based ester and other insulating oils: Commercial natural ester, synthetic ester, and mineral oil. Biomass Convers. Biorefinery.

[55] Dang V.H., Beroual A., Perrier C. (2012). Comparative study of statistical breakdown in mineral, synthetic and natural ester oils under AC voltage. IEEE Trans. Dielectr. Electr. Insul..

[56] Gengadevi K., .Madavan R. (2024). Analysis on effect of ultrasonic process on non-edible ester oils physiochemical properties. Energy Sources Part A Recover. Util. Environ. Eff..

[57] Tharamal L., Preetha P., Sindhu T.K. (2022). Assessing the Degradation of Transformer Oil From Partial Discharge Measurement Data Using Histogram Similarity Measures. IEEE Trans. Instrum. Meas..

[58] Subburaj S.K., Rengaraj M., Mariappan R. (2020). Evaluating critical characteristics of vegetable oil as a biodegradable insulating oil for transformer. Int. J. Emerg. Electr. Power Syst..

[59] Primo V.A., García B., Burgos J.C., Pérez D. (2020). AC breakdown voltage of Fe_3_O_4_ based nanodielectric fluids. Part 2: Analysis of fluids with high moisture content. IEEE Trans. Dielectr. Electr. Insul..

[60] (2018). Insulating Liquids—Determination of the Breakdown Voltage at Power Frequency—Test Method.

[61] (2004). Insulating Liquids—Measurement of Relative Permittivity, Dielectric Dissipation Factor (*tanδ*) and d.c. Resistivity.

[62] (2020). Insulating Liquids—Oil-Impregnated Paper and Pressboard—Determination of Water by Karl Fischer Titration.

[63] (2023). Insulating Liquids—Determination of Acidity—Automatic Potentiometric Titration.

[64] (2023). Standard Test Method for Flash and Fire Points by Cleveland Open Cup Tester.

[65] (2023). Standard Test Methods for Flash Point by Pensky-Martens Closed Cup Tester.

[66] Abdali A., Abedi A., Mazlumi K., Rabiee A., Guerrero J.M. (2023). Precise thermo-fluid dynamics analysis of corrugated wall distribution transformer cooled with mineral oil-based nanofluids: Experimental verification. Appl. Therm. Eng..

[67] Altay R., Santisteban A., Olmo C., Delgado F., Renedo C.J., Köseoğlu A., Ortiz A. Performance analysis of natural, synthetic and mineral oil in a 100 MVA power transformer. Proceedings of the 2020 IEEE 3rd International Conference on Dielectrics (ICD).

[68] Sitorus H.B., Setiabudy R., Bismo S., Beroual A. (2016). Jatropha curcas methyl ester oil obtaining as vegetable insulating oil. IEEE Trans. Dielectr. Electr. Insul..

[69] Alizadeh H., Pourpasha H., Zeinali Heris S., Estellé P. (2022). Experimental investigation on thermal performance of covalently functionalized hydroxylated and non-covalently functionalized multi-walled carbon nanotubes/transformer oil nanofluid. Case Stud. Therm. Eng..

[70] Maiti P.K., Chakraborty M., Kumar M., Nasakar M. Evaluation of Blended Mineral and Ester Oil Nanofluids for Transformer Applications. Proceedings of the 2024 IEEE 7th International Conference on Condition Assessment Techniques in Electrical Systems (CATCON).

[71] Sima W., Shi J., Yang Q., Huang S., Cao X. (2015). Effects of conductivity and permittivity of nanoparticle on transformer oil insulation performance: Experiment and theory. IEEE Trans. Dielectr. Electr. Insul..

[72] Danikas M. (2018). Breakdown in Nanofluids: A Short Review on Experimental Results and Related Mechanisms. Eng. Technol. Appl. Sci. Res..

[73] Lee J.C., Seo H.S., Kim Y.J. (2012). The increased dielectric breakdown voltage of transformer oil-based nanofluids by an external magnetic field. Int. J. Therm. Sci..

[74] Zhang C., Wang Y., Yan Z., He Z. (2019). Interplay between nanoparticles and water on dielectric properties of nanofluids. IEEE Trans. Dielectr. Electr. Insul..

[75] Samy A.M., Ibrahim M.E., Abd-Elhady A.M., Izzularab M.A. (2020). On electric field distortion for breakdown mechanism of nanofilled transformer oil. Int. J. Electr. Power Energy Syst..

[76] Sergis A., Hardalupas Y. (2011). Anomalous heat transfer modes of nanofluids: A review based on statistical analysis. Nanoscale Res. Lett..

[77] Khan S.A., Khan A.A., Tariq M. Experimental Analysis for the Effect of Fe/Ti Oxides and Fe-Cu Nanoparticles on the Dielectric Strength of Transformer Oil. Proceedings of the 2019 International Conference on High Voltage Engineering and Technology (ICHVET).

[78] Chakraborty B., Raj K.Y., Pradhan A.K., Chatterjee B., Chakravorti S., Dalai S. (2021). Investigation of Dielectric Properties of TiO_2_ and Al_2_O_3_ nanofluids by Frequency Domain Spectroscopy at Different Temperatures. J. Mol. Liq..

[79] Hou J., Wang L., Wang C., Zhang S., Liu H., Li S., Wang X. (2019). Toxicity and mechanisms of action of titanium dioxide nanoparticles in living organisms. J. Environ. Sci..

[80] Wanatasanappan V.V., Rezman M., Abdullah M.Z. (2022). Thermophysical Properties of Vegetable Oil-Based Hybrid Nanofluids Containing Al_2_O_3_-TiO_2_ Nanoparticles as Insulation Oil for Power Transformers. Nanomaterials.

[81] Mansour D.E.A., Shaalan E.M., Ward S.A., El Dein A.Z., Karaman H.S., Ahmed H.M. (2019). Multiple nanoparticles for improvement of thermal and dielectric properties of oil nanofluids. IET Sci. Meas. Technol..

[82] Khaled U., Beroual A., Khan Y. (2019). Statistical investigation of AC breakdown voltage of natural ester with electronic scavenger additives. IEEE Trans. Dielectr. Electr. Insul..

[83] Khaled U., Beroual A. (2019). Statistical Investigation of AC Dielectric Strength of Natural Ester Oil-Based Fe_3_O_4_, Al_2_O_3_, and SiO_2_ Nano-Fluids. IEEE Access.

[84] Koutras K.N., Naxakis I.A., Antonelou A.E., Charalampakos V.P., Pyrgioti E.C., Yannopoulos S.N. (2020). Dielectric strength and stability of natural ester oil based TiO_2_ nanofluids. J. Mol. Liq..

[85] Farade R.A., Wahab N.I.B.A., Mansour D.E.A., Azis N.B., Jasni J., Banapurmath N.R., Soudagar M.E.M. (2020). Investigation of the Dielectric and Thermal Properties of Non-Edible Cottonseed Oil by Infusing h-BN Nanoparticles. IEEE Access.

[86] Sumathi S., Rajesh R. (2020). Improvement on the characteristics of transformer oil using nanofluids. Curr. Sci..

[87] Raja S., Koperundevi G. (2020). Titania-based transformer nanofluid: A study on the synthesis for enhanced breakdown strength and its humidity ageing. IET Nanodielectrics.

[88] Almeida C., Paul S., Godson Asirvatham L., Manova S., Nimmagadda R., Raja Bose J., Wongwises S. (2020). Experimental Studies on Thermophysical and Electrical Properties of Graphene–Transformer Oil Nanofluid. Fluids.

[89] Olmo C., Fernandez I., Santisteban A., Mendez C., Ortiz F., Ortiz A. Effect of maghemite nanoparticles on insulation and cooling behaviour of a natural ester used in power transformers. Proceedings of the 2019 IEEE 20th International Conference on Dielectric Liquids (ICDL).

[90] Abid M.A., Khan I., Ullah Z., Ullah K., Haider A., Ali S.M. (2019). Dielectric and Thermal Performance Up-Gradation of Transformer Oil Using Valuable Nano-Particles. IEEE Access.

[91] Khaled U., Beroual A. (2019). AC dielectric strength of synthetic ester-based Fe_3_O_4_, Al_2_O_3_ and SiO_2_ nanofluids—Conformity with normal and weibull distributions. IEEE Trans. Dielectr. Electr. Insul..

[92] Hanai M., Hosomi S., Kojima H., Hayakawa N., Okubo H. Dependence of TiO_2_ and ZnO nanoparticle concentration on electrical insulation characteristics of insulating oil. Proceedings of the 2013 Annual Report Conference on Electrical Insulation and Dielectric Phenomena.

[93] Ge Y., Niu M., Wang L., Huang M., Lv Y., Li C., Yuan J. Effects of TiO_2_ Nanoparticle Size on Dielectric Properties of Transformer Oil. Proceedings of the 2018 IEEE Conference on Electrical Insulation and Dielectric Phenomena (CEIDP).

[94] Mansour D.E.A., Atiya E.G., Khattab R.M., Azmy A.M. Effect of titania nanoparticles on the dielectric properties of transformer oil-based nanofluids. Proceedings of the 2012 Annual Report Conference on Electrical Insulation and Dielectric Phenomena.

[95] Rafiq M., Li C., Ge Y., Lv Y., Yi K. Effect of Fe_3_O_4_ nanoparticle concentrations on dielectric property of transformer oil. Proceedings of the 2016 IEEE International Conference on High Voltage Engineering and Application (ICHVE).

[96] Atiya E.G., Mansour D.E.A., Khattab R.M., Azmy A.M. (2015). Dispersion behavior and breakdown strength of transformer oil filled with TiO2 nanoparticles. IEEE Trans. Dielectr. Electr. Insul..

[97] Khaled U., Beroual A. Comparative Study on the AC Breakdown Voltage of Transformer Mineral Oil with Transformer Oil-based Al_2_O_3_ Nanofluids. Proceedings of the 2018 IEEE International Conference on High Voltage Engineering and Application (ICHVE).

[98] Rajda N., Patel R.R. Characterization of virgin and aged transformer oil with SiO_2_ nanoparticles. Proceedings of the 2017 Innovations in Power and Advanced Computing Technologies (i-PACT).

[99] Lv Y.Z., Li C., Sun Q., Huang M., Li C.R., Qi B. (2016). Effect of dispersion method on stability and dielectric strength of transformer oil-based TiO_2_ nanofluids. Nanoscale Res. Lett..

[100] Muangpratoom P., Kunakorn A., Pattanadech N., Vittayakorn W., Thungsook K. Dielectric Properties of Mineral Oil-based Nanofluids using Zinc Oxide Nano-composites for Power Transformer Application. Proceedings of the 2018 Condition Monitoring and Diagnosis (CMD).

[101] Ibrahim M.E., Abd-Elhady A.M., Izzularab M.A. (2016). Effect of nanoparticles on transformer oil breakdown strength: Experiment and theory. IET Sci. Meas. Technol..

[102] Rafiq M., Li C., Du Q., Lv Y., Yi K. Effect of SiO_2_ nanoparticle on insulating breakdown properties of transformer oil. Proceedings of the 2016 IEEE International Conference on High Voltage Engineering and Application (ICHVE).

[103] Du Y.F., Lv Y.Z., Zhou J.Q., Li X.X., Li C.R. Breakdown properties of transformer oil-based TiO_2_ nanofluid. Proceedings of the 2010 Annual Report Conference on Electrical Insulation and Dielectic Phenomena.

[104] Duzkaya H., Beroual A. (2021). Statistical Analysis of AC Dielectric Strength of Natural Ester-Based ZnO Nanofluids. Energies.

[105] Yadav N., Jarial R.K., Rao U.M. (2018). Characterization of Mineral oil Based Fe_3_O_4_ Nanofluid for Application in Oil Filled Transformers. Int. J. Electr. Eng. Informatics.

[106] Khan S.A., Khan S.A., Masood A., Zuberi M.U., Khan A.A. Nanofluidic Experimentation: Transforming Natural Esters into Enhanced Dielectric Fluids. Proceedings of the 2024 3rd International conference on Power Electronics and IoT Applications in Renewable Energy and its Control (PARC).

[107] Abd-Elhady A.M., Ibrahim M.E., Taha T., Izzularab M.A. (2018). Effect of temperature on AC breakdown voltage of nanofilled transformer oil. IET Sci. Meas. Technol..

[108] Palanisamy K.V., Subramaniam C., Sakthivel B. (2022). Evaluating the role of carbon quantum dots covered silica nanofillers on the partial discharge performance of transformer insulation. Turk. J. Electr. Eng. Comput. Sci..

[109] Atiya E.G., Mansour D.E.A., Izzularab M.A. Partial Discharge Activity of Al_2_O_3_ Nanofluid Impregnated Paper Insulation System. Proceedings of the 2020 International Symposium on Electrical Insulating Materials (ISEIM).

[110] Atiya E.G., Mansour D.E.A., Izzularab M.A. (2020). Partial Discharge Development in Oil-Based Nanofluids: Inception, Propagation and Time Transition. IEEE Access.

[111] Balaji S., Salem T., Chandrasekar I.S. (2024). Impact of Harmonic Voltage on the Partial Discharge Properties of Eco-Friendly Nanofluid. J. Environ. Nanotechnol.

[112] Kurimskỳ J., Rajňák M., Paulovičová K., Šárpataky M. (2024). Electric partial discharges in biodegradable oil-based ferrofluids: A study on effects of magnetic field and nanoparticle concentration. Heliyon.

[113] Jin H., Morshuis P., Mor A.R., Smit J.J., Andritsch T. (2015). Partial discharge behavior of mineral oil based nanofluids. IEEE Trans. Dielectr. Electr. Insul..

[114] Fal J., Mahian O., Zyla G. (2018). Nanofluids in the Service of High Voltage Transformers: Breakdown Properties of Transformer Oils with Nanoparticles, a Review. Energies.

[115] Oparanti S., Fofana I., Jafari R., Zarrougui R. (2024). A state-of-the-art review on green nanofluids for transformer insulation. J. Mol. Liq..

[116] Fahmi D., Akbar M.F., Negara I., Hernanda I., Asfani D.A., Zaidan R.A., Fadhilah A. (2025). Influence of metal particles shape on direct current voltage electric properties of nanofluids. Int. J. Electr. Comput. Eng. (2088-8708).

[117] Suresha C., Rudranna N., Khan F.A. Effect of Nano particles in improvement of dielectric characteristics of liquid insulation. Proceedings of the 2023 IEEE Electrical Insulation Conference (EIC).

[118] Karatas M., Bicen Y. (2022). Nanoparticles for next-generation transformer insulating fluids: A review. Renew. Sustain. Energy Rev..

[119] Maiti P.K. (2023). Behavior of Nanoparticles in Service Transformer Oils and their Performance on Laboratory Ageing. Power Res.-A J. CPRI.

[120] Hiremath V.K., Muduli R.C., Kale P. (2023). Investigation of the Stability and Insulating Properties of Mineral Oil-Based Surface Modified Silicon Nanofluid. IEEE Trans. Dielectr. Electr. Insul..

[121] Thomas P., Hudedmani N.E., Prasath R.T.A.R., Roy N.K., Mahato S.N. (2019). Synthetic ester oil based high permittivity CaCu3Ti4O12 (CCTO) nanofluids an alternative insulating medium for power transformer. IEEE Trans. Dielectr. Electr. Insul..

[122] Amalanathan A.J., Sarathi R., Zdanowski M. (2023). A Critical Overview of the Impact of Nanoparticles in Ester Fluid for Power Transformers. Energies.

[123] Ghoneim S., Dessouky S., Taha I., Shaban M., Abdelwahab S.A.M. (2021). A new approach of tap changer maintenance incorporating nanoparticle insulating oil. Electr. Eng..

[124] Mentlik V., Trnka P., Hornak J., Totzauer P. (2018). Development of a Biodegradable Electro-Insulating Liquid and Its Subsequent Modification by Nanoparticles. Energies.

[125] Khairul M., Shah K., Doroodchi E., Azizian R., Moghtaderi B. (2016). Effects of surfactant on stability and thermo-physical properties of metal oxide nanofluids. Int. J. Heat Mass Transf..

[126] Yu D., Zhengyong H., Bin D., Gang C., Tianqin L., Jian L. Analysis of Thermal Conductivity Calculation Model of Nano Insulating Oil. Proceedings of the 2020 IEEE International Conference on High Voltage Engineering and Application (ICHVE).

[127] Taha-Tijerina J., PeÑa N.C.D.L., Cue-Sampedro R., Rivera-Solorio C. (2019). Thermo-physical evaluation of dielectric mineral oil-based nitride and oxide nanofluids for thermal transport applications. J. Therm. Sci. Technol..

[128] Singh M., Kundan L. (2013). Experimental study on thermal conductivity and viscosity of Al_2_O_3_-nanotransformer oil. Int. J. Theor. Appl. Res. Mech. Eng. (IJTARME).

[129] Beheshti A., Shanbedi M., Heris S.Z. (2014). Heat transfer and rheological properties of transformer oil-oxidized MWCNT nanofluid. J. Therm. Anal. Calorim..

[130] Wang B., Li J., Du B., Zhang Z. Study on the stability and viscosity of Fe_3_O_4_ nano-particles vegetable insulating oils. Proceedings of the 2012 International Conference on High Voltage Engineering and Application.

[131] Fernández I., Valiente R., Ortiz F., Renedo C.J., Ortiz A. (2020). Effect of TiO_2_ and ZnO Nanoparticles on the Performance of Dielectric Nanofluids Based on Vegetable Esters During Their Aging. Nanomaterials.

[132] Swati K., Vishnu M., Prakash K.A., Sarathi R. (2020). Investigation on heat transfer characteristics of nano titania added transformer oil with hotspot temperature. Nano Express.

[133] Swift G., Zocholl E., Bajpai M., Burger J., Castro C., Chano S., Cobelo F., de Sa P., Fennell E., Gilbert J. (2001). Adaptive transformer thermal overload protection. IEEE Trans. Power Deliv..

[134] Shukla G., Aiyer H. (2015). Thermal conductivity enhancement of transformer oil using functionalized nanodiamonds. IEEE Trans. Dielectr. Electr. Insul..

[135] Estellé P., Cabaleiro D., Żyła G., Lugo L., Murshed S.S. (2018). Current trends in surface tension and wetting behavior of nanofluids. Renew. Sustain. Energy Rev..

[136] Choi C., Yoo H., Oh J. (2008). Preparation and heat transfer properties of nanoparticle-in-transformer oil dispersions as advanced energy-efficient coolants. Curr. Appl. Phys..

[137] Liu D., Zhou Y., Yang Y., Zhang L., Jin F. (2016). Characterization of high performance AIN nanoparticle-based transformer oil nanofluids. IEEE Trans. Dielectr. Electr. Insul..

[138] Jin H., Andritsch T., Tsekmes I.A., Kochetov R., Morshuis P.H., Smit J.J. (2014). Properties of Mineral Oil based Silica Nanofluids. IEEE Trans. Dielectr. Electr. Insul..

[139] Thomas P., Hudedmani N.E. (2019). The effect of nanoparticles on the insulating characteristics of synthetic esters. Proceedings of the 2019 International Conference on High Voltage Engineering and Technology (ICHVET).

[140] Dombek G., Nadolny Z., Przybyłek P. The study of thermal properties of mineral oil and synthetic ester modified by nanoparticles TiO_2_ and C60. Proceedings of the 2014 ICHVE International Conference on High Voltage Engineering and Application.

[141] Jacob J., Preetha P., Sindhu T.K. (2020). Stability analysis and characterization of natural ester nanofluids for transformers. IEEE Trans. Dielectr. Electr. Insul..

[142] Wang J., Yang X., KlemeÅ J.J., Tian K., Ma T., Sunden B. (2023). A review on nanofluid stability: Preparation and application. Renew. Sustain. Energy Rev..

[143] Borzuei M., Baniamerian Z. (2020). Role of nanoparticles on critical heat flux in convective boiling of nanofluids: Nanoparticle sedimentation and Brownian motion. Int. J. Heat Mass Transf..

[144] Fujiwara K., Daimo M., Ueki Y., Ohara T., Shibahara M. (2019). Thermal conductivity of nanofluids: A comparison of EMD and NEMD calculations. Int. J. Heat Mass Transf..

[145] Verwey E.J.W. (1947). Theory of the Stability of Lyophobic Colloids. J. Phys. Colloid Chem..

[146] Ding J., Zhao H., Yu H. (2021). Graphene nanofluids based on one-step exfoliation and edge-functionalization. Carbon.

[147] Dong M., Shen L.P., Wang H., Wang H.B., Miao J. (2013). Investigation on the Electrical Conductivity of Transformer Oil-Based AlN Nanofluid. J. Nanomater..

[148] Chiesa M., Das S.K. (2009). Experimental investigation of the dielectric and cooling performance of colloidal suspensions in insulating media. Colloids Surfaces A Physicochem. Eng. Asp..

[149] Mansour D.A., Elsaeed A.M. Heat transfer properties of transformer oil-based nanofluids filled with Al_2_O_3_ nanoparticles. Proceedings of the 2014 IEEE International Conference on Power and Energy (PECon).

[150] Amici J., Allia P., Tiberto P., Sangermano M. (2011). Poly(ethylene glycol)-Coated Fe_3_O_4_ Nanoparticles by UV-Thiol-Ene Addition of PEG Dithiol on Vinyl-Functionalized Magnetite Surface. Macromol. Chem. Phys..

[151] Hwang Y., Park H., Lee J., Jung W. (2006). Thermal conductivity and lubrication characteristics of nanofluids. Curr. Appl. Phys..

[152] Karthik R., Cavallini A., Azcarraga C. Investigations on the effect of nanoparticles in mineral oil. Proceedings of the 2014 IEEE Conference on Electrical Insulation and Dielectric Phenomena (CEIDP).

[153] Given M.J., Wilson M.P., McGlone P., Timoshkin I.V., Wang T., MacGregor S.J., Lehr J.M. The influence of magnetite nano particles on the behaviour of insulating oils for pulse power applications. Proceedings of the 2011 Annual Report Conference on Electrical Insulation and Dielectric Phenomena.

[154] Primo V.A., García B., Burgos J.C., Pérez D. (2019). Evaluation of the Stability of Dielectric Nanofluids for Use in Transformers under Real Operating Conditions. Nanomaterials.

[155] Bhunia M.M., Panigrahi K., Naskar C.B., Bhattacharjee S., Chattopadhyay K.K., Chattopadhyay P. (2021). 2D square nanosheets of Anatase TiO2: A surfactant free nanofiller for transformer oil nanofluids. J. Mol. Liq..

[156] Ch Shill D., Das A.K., Chatterjee S. Insulation and Cooling Performance of Transformer Oil Based Nanofluid. Proceedings of the 2020 International Conference on Computer, Electrical & Communication Engineering (ICCECE).

[157] Mohamad M.S., Zainuddin H., Ab Ghani S., Chairul I.S. (2017). AC breakdown voltage and viscosity of palm fatty acid ester (PFAE) oil-based nanofluids. J. Electr. Eng. Technol..

[158] Chen J., Sun P., Sima W., Shao Q., Ye L., Li C. (2019). A Promising Nano-Insulating-Oil for Industrial Application: Electrical Properties and Modification Mechanism. Nanomaterials.

[159] Li S., Karlsson M., Liu R., Ahniyaz A., Fornara A., Salazar-Sandoval E.J. The effect of ceria nanoparticles on the breakdown strength of transformer oil. Proceedings of the 2015 IEEE 11th International Conference on the Properties and Applications of Dielectric Materials (ICPADM).

[160] Viali W.R., Alcântara G.B., Sartoratto P.P.C., Soler M.A.G., Mosiniewicz-Szablewska E., Andrzejewski B., de Morais P.C. (2010). Investigation of the Molecular Surface Coating on the Stability of Insulating Magnetic Oils. J. Phys. Chem. C.

[161] Zargartalebi M., Azaiez J. (2018). Heat transfer analysis of nanofluid based microchannel heat sink. Int. J. Heat Mass Transfer.

[162] Mostafizur R., Rasul M., Nabi M. (2022). Effect of surfactant on stability, thermal conductivity, and viscosity of aluminium oxide–methanol nanofluids for heat transfer applications. Therm. Sci. Eng. Prog..

[163] Yu H., Hermann S., Schulz S.E., Gessner T., Dong Z., Li W.J. (2012). Optimizing sonication parameters for dispersion of single-walled carbon nanotubes. Chem. Phys..

[164] Aseyev V.O., Tenhu H., Winnik F.M., Khokhlov A.R. (2006). Temperature Dependence of the Colloidal Stability of Neutral AmphiphilicPolymers in Water. Conformation-Dependent Design of Sequences in Copolymers II.

[165] Shanbedi M., Zeinali Heris S., Maskooki A. (2015). Experimental investigation of stability and thermophysical properties of carbon nanotubes suspension in the presence of different surfactants. J. Therm. Anal. Calorim..

[166] Sadri R., Ahmadi G., Togun H., Dahari M., Kazi S.N., Sadeghinezhad E., Zubir N. (2014). An experimental study on thermal conductivity and viscosity of nanofluids containing carbon nanotubes. Nanoscale Res. Lett..

[167] Tian S., Gao W., Liu Y., Kang W., Yang H. (2020). Effects of surface modification Nano-SiO_2_ and its combination with surfactant on interfacial tension and emulsion stability. Colloids Surfaces A Physicochem. Eng. Asp..

[168] Ken D.S., Sinha A. (2020). Recent developments in surface modification of nano zero-valent iron (nZVI): Remediation, toxicity and environmental impacts. Environ. Nanotechnol. Monit. Manag..

[169] Caprara A., Ciotti G., Melloni M. A Novel On-Line Monitoring System for Diagnostics Parameters of EHV GIS Electrical Apparatuses of Transmission Networks. Proceedings of the 2020 International Symposium on Electrical Insulating Materials (ISEIM).

[170] Du Y., Lv Y., Li C., Zhong Y., Chen M., Zhang S., Zhou Y., Chen Z. (2012). Effect of water adsorption at nanoparticle–oil interface on charge transport in high humidity transformer oil-based nanofluid. Colloids Surfaces A Physicochem. Eng. Asp..

[171] Sun Z., Ge Y., Lv Y., Huang M., Li C., Du Y. The effects of TiO_2_ nanoparticles on insulation and charge transport characteristics of aged transformer oil. Proceedings of the 2019 IEEE 20th International Conference on Dielectric Liquids (ICDL).

[172] De Y., Lv Y., Zhou J., Chen M., Li X., Li C. Effect of Ageing on Insulating Property of Mineral Oil-based TiO_2_ Nanofluids. Proceedings of the 2011 IEEE International Conference on Dielectric Liquids.

[173] Zhang J., Wang F., Li J., Ran H., Li X., Fu Q. (2017). Breakdown Voltage and Its Influencing Factors of Thermally Aged Oil-Impregnated Paper at Pulsating DC Voltage. Energies.

[174] Liang N., Liao R., Xiang M., Mo Y., Yuan Y. (2018). Effect of Nano Al_2_O_3_ Doping on Thermal Aging Properties of Oil-Paper Insulation. Energies.

[175] Maharana M., Baruah N., Nayak S.K., Sahoo N. Comparative study of mechanical and electrical strength of kraft paper in nanofluid based transformer oil and mineral oil. Proceedings of the 2017 International Symposium on Electrical Insulating Materials (ISEIM).

[176] (2000). High-Voltage Test Techniques—Partial Discharge Measurements.

[177] (2008). 2008—Plastics—Determination of the Thermal Conductivity and Thermal Diffusivity—Part 2: Laser Flash Method.

[178] (2023). Standard Test Method for Kinematic Viscosity of Transparent and Opaque Liquids (and Calculation of Dynamic Viscosity).

[179] (2012). Insulating Liquids—Test Methods for the Determination of the Oxidation Stability of Insulating Oils.

